# Macrophage ATF6 accelerates corticotomy-assisted orthodontic tooth movement through promoting Tnfα transcription

**DOI:** 10.1038/s41368-025-00359-7

**Published:** 2025-04-01

**Authors:** Zhichun Jin, Hao Xu, Weiye Zhao, Kejia Zhang, Shengnan Wu, Chuanjun Shu, Linlin Zhu, Yan Wang, Lin Wang, Hanwen Zhang, Bin Yan

**Affiliations:** 1 Department of Orthodontics, The Affiliated Stomatological Hospital of Nanjing Medical University, Nanjing, China; 2 State Key Laboratory Cultivation Base of Research, Prevention and Treatment for Oral Diseases, Nanjing Medical University, Nanjing, China; 3 Jiangsu Province Engineering Research Center of Stomatological Translational Medicine, Nanjing, China; 4 Department of Bioinformatics, School of Biomedical Engineering and Informatics, Nanjing Medical University, Nanjing, China; 5 School of Basic Medical Sciences, Nanjing Medical University, Nanjing, China; 6 Key Laboratory of Targeted Intervention of Cardiovascular Disease, Collaborative Innovation Center for Cardiovascular Disease Translational Medicine, Nanjing Medical University, Nanjing, China

**Keywords:** Bone, Stress signalling

## Abstract

Corticotomy is a clinical procedure to accelerate orthodontic tooth movement characterized by the regional acceleratory phenomenon (RAP). Despite its therapeutic effects, the surgical risk and unclear mechanism hamper the clinical application. Numerous evidences support macrophages as the key immune cells during bone remodeling. Our study discovered that the monocyte-derived macrophages primarily exhibited a pro-inflammatory phenotype that dominated bone remodeling in corticotomy by CX3CR1^CreERT2^; R26^GFP^ lineage tracing system. Fluorescence staining, flow cytometry analysis, and western blot determined the significantly enhanced expression of binding immunoglobulin protein (BiP) and emphasized the activation of sensor activating transcription factor 6 (ATF6) in macrophages. Then, we verified that macrophage specific ATF6 deletion (ATF6^f/f^; CX3CR1^CreERT2^ mice) decreased the proportion of pro-inflammatory macrophages and therefore blocked the acceleration effect of corticotomy. In contrast, macrophage ATF6 overexpression exaggerated the acceleration of orthodontic tooth movement. In vitro experiments also proved that higher proportion of pro-inflammatory macrophages was positively correlated with higher expression of ATF6. At the mechanism level, RNA-seq and CUT&Tag analysis demonstrated that ATF6 modulated the macrophage-orchestrated inflammation through interacting with *Tnfα* promotor and augmenting its transcription. Additionally, molecular docking simulation and dual-luciferase reporter system indicated the possible binding sites outside of the traditional endoplasmic reticulum-stress response element (ERSE). Taken together, ATF6 may aggravate orthodontic bone remodeling by promoting *Tnfα* transcription in macrophages, suggesting that ATF6 may represent a promising therapeutic target for non-invasive accelerated orthodontics.

## Introduction

Corticotomy is a clinical procedure to accelerate orthodontic tooth movement (OTM) through inducing the regional acceleratory phenomenon (RAP), which is characterized by acceleration of remodeling activities after surgical incision in bone.^1^ It shortens the long orthodontic treatment duration and helps to minimize complications.^2^ While this highlights the procedure’s clinical relevance, molecular mechanisms of RAP remain largely undefined.^3,4^ An in-depth investigation into the mechanisms of corticotomy will provide a theoretical basis for the application of corticotomy and future non-invasive accelerated techniques, and also for expanding into the broader orthopedic field, including treatments for fractures and arthritis.

Cumulative evidence has emphasized the critical role of macrophages in bone remodeling, sparking a keen interest in their implications for OTM.^5,6^ Macrophages in alveolar bone react to various damage-associated molecular patterns (DAMPs) and undergo differentiation into distinct phenotypes.^7^ Previous studies from our group and others have demonstrated that the pro-inflammatory macrophage polarization accelerates bone resorption and subsequent OTM through secretion of diverse cytokines.^8,9^ However, the origins of macrophages and the molecular mechanisms driving pro-inflammatory macrophage actions induced by corticotomy remain to be fully elucidated.

Macrophage functions, such as antigen presentation and cytokine secretion dependent on protein synthesis,^10^ which is a requisite for proteome integrity and optimal functionality of cells. The endoplasmic reticulum (ER) plays the pivotal role in protein synthesis. However, factors such as inflammation, hypoxia, and nutrient deficiency can cause the accumulation of unfolded and misfolded proteins within the ER, leading to ER stress and subsequent unfolded protein response (UPR) with an aim to restore cellular homeostasis.^11,12^ Nevertheless, the activation and function of the UPR within the corticotomy microenvironment are underexplored.

The UPR is orchestrated by the synergistic actions of activating transcription factor 6 (ATF6), inositol-requiring enzyme 1 α (IRE1α), and protein kinase RNA-like ER kinase (PERK) pathways.^13^ ATF6, uniquely among the three ER membrane sensor proteins, functions as a transcription factor.^14^ Upon release of physically bound immunoglobulin-binding protein (BiP) from the ER lumen, unbounded ATF6 translocates to the Golgi apparatus where it is sequential cleavage by site 1 (S1P) and site 2 (S2P) proteases, yielding the active transcription factor (ATF6 p50).^15^ The liberated cytosolic N-terminal portion of ATF6 then migrates to the nucleus and binds to the ER-stress response element (ERSE) to activate gene expression of ER chaperones and ER-associated protein degradation (ERAD) components.^16^ ATF6 has been shown to be instrumental in regulating macrophage functions across various tissues.^17^ For instance, ATF6 mediates the pro-inflammatory enhancement of TLR4 response and cytokine production in liver Kupffer cells.^18^ Moreover, deficiency of microglial ATF6 in autoimmune encephalomyelitis has been reported to mitigate inflammatory responses associated with the degradation of p65.^19^ However, there’s limited evidence of ATF6 orchestrating macrophage inflammation, particularly in the context of bone remodeling. Therefore, we hypothesize that macrophage ATF6 might be critical for driving alveolar bone remodeling in response to corticotomy.

Here, we explore the potential involvement of macrophage ATF6 activation in corticotomy-assisted orthodontic tooth movement. This acceleration is due to the elevated pro-inflammatory response and Tnfα production. Our findings offer novel molecular understanding of the accelerated orthodontics and may help facilitate the development of future non-invasive interventions.

## Results

### Monocyte-derived macrophages in favor of pro-inflammatory phenotype in corticotomy

**Fig. 1:**
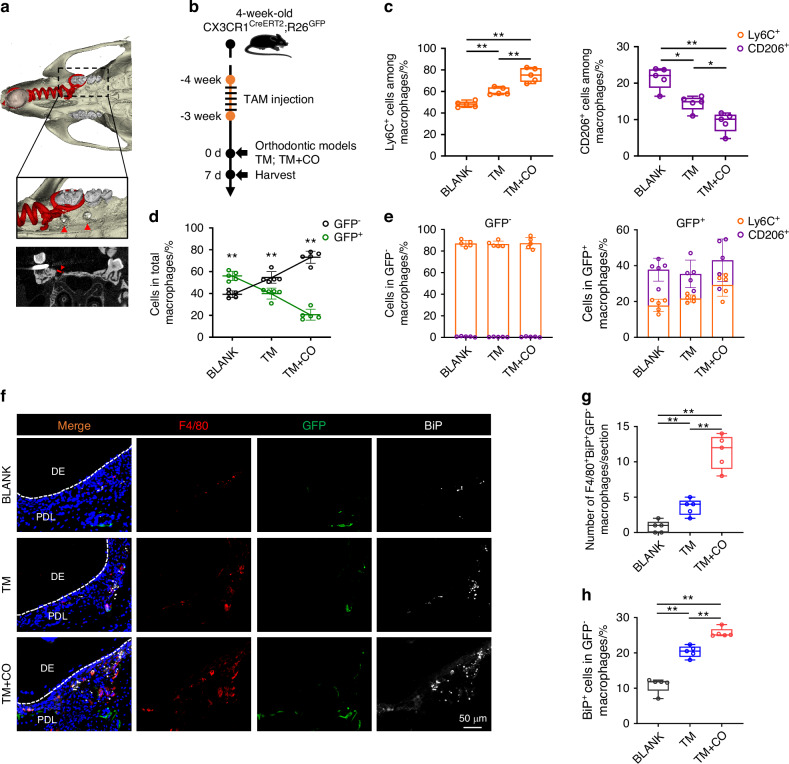
Monocyte-derived macrophages in favor of pro-inflammatory phenotype in corticotomy. **a** Representative 3D reconstruction images show the modeling method of TM + CO group. **b** Experimental design for tamoxifen‐treated CX3CR1^CreERT2^; R26^GFP^ mice to deplete GFP^+^ monocyte-derived macrophages. **c** FACS analysis of CD45^+^F4/80^+^CD11b^+^Ly6C^+^ pro-inflammatory macrophages and CD45^+^F4/80^+^CD11b^+^CD206^+^ reparative macrophages in murine periodontal tissues from BLANK, TM and TM + CO groups. **d** FACS analysis of GFP^+^CD45^+^F4/80^+^CD11b^+^ resident and GFP^−^CD45^+^F4/80^+^CD11b^+^ monocyte-derived macrophages in murine periodontal tissues from BLANK, TM and TM + CO groups. **e** FACS analysis of CD45^+^F4/80^+^CD11b^+^Ly6C^+^ pro-inflammatory macrophages and CD45^+^F4/80^+^CD11b^+^CD206^+^ reparative macrophages in GFP^−^ monocyte-derived macrophages or GFP^+^ resident macrophages in murine periodontal tissues from BLANK, TM and TM + CO groups. **f**, **g** Representative immunofluorescence staining images and quantification of F4/80^+^ (red) BiP^+^ (white) GFP^−^ cells in murine periodontal tissues. Scale bars, 50 μm. **h** FACS analysis of BiP^+^ macrophages in GFP^−^CD45^+^F4/80^+^CD11b^+^ monocyte-derived macrophages in murine periodontal tissues from BLANK, TM and TM + CO groups. Data represent mean ± SD. N = 5. *P < 0.05, **P < 0.01. TM tooth movement, TM + CO corticotomy-assisted orthodontic tooth movement

To investigated the role of macrophages in corticotomy, we first conducted murine models of orthodontic tooth movement (TM) or corticotomy-assisted orthodontic tooth movement (TM + CO) (Fig. 1a). Micro‐CT analysis showed that corticotomy significantly accelerated tooth movement, and the velocity of tooth movement reached the peak at Day 7 (Fig. S1a, b). Histological staining of maxillary first molars revealed that corticotomy increased the number of TRAP^+^ osteoclasts and promoted alveolar bone remodeling in response to local inflammation (Fig. S1c, d). Further, pro‐inflammatory macrophage phenotypes (iNOS^+^F4/80^+^) and restorative macrophage phenotypes (CD206^+^F4/80^+^) in the indicated sites were identified by immunofluorescence staining, which showed that corticotomy led to an increase of iNOS^+^F4/80^+^ cells and a decrease of CD206^+^F4/80^+^ cells in periodontal tissues (Fig. S1e–g).

To determine whether the accumulation of pro‐inflammatory macrophages was dependent on recruited macrophages or resident macrophages, we performed a CX3CR1-based lineage tracing approach as previously described.^20^ Since the chemokine receptor CX3CR1 is highly expressed in both monocyte-derived and resident macrophages,^21,22^ tamoxifen was injected into 4-week-old CX3CR1^CreERT2^; R26^GFP^ mice to induce the expression of Cre recombinase and labeled monocyte-derived and resident macrophages as GFP^+^. After three weeks, monocyte-derived macrophages, replaced by bone marrow-derived monocytes, were GFP^−^, while the self-renewable tissue-resident macrophages remained GFP^+^.^20,23^ Orthodontic models were then established to differentiate between monocyte-derived and resident macrophages (Figs. 1b and S2a). First, FACS analysis of CD45^+^CD11b^+^F4/80^+^Ly6C^+^ pro‐inflammatory macrophages and CD45^+^CD11b^+^F4/80^+^CD206^+^ restorative macrophages relative to the total macrophages in murine periodontal tissues reciprocated the immunostaining findings above (Figs. 1c and S2b). Moreover, we also observed an elevated proportion of GFP^−^CD45^+^CD11b^+^F4/80^+^ monocyte-derived macrophages in periodontal tissues after corticotomy, while GFP^+^CD45^+^CD11b^+^F4/80^+^ resident macrophages declined (Fig. 1d).

After identifying the origins of accumulated macrophages in periodontal tissues, we set out to investigate the functional diversity of macrophages from different origins. FACS analysis showed that GFP^−^CD45^+^CD11b^+^F4/80^+^ monocyte-derived macrophages mainly polarized towards Ly6C^+^ pro-inflammatory phenotype while GFP^+^CD45^+^CD11b^+^F4/80^+^ tissue resident macrophages exhibited more CD206^+^ restorative character in periodontal tissues from CX3CR1^CreERT2^; R26^GFP^ mice (Fig. 1e). These results reflect that pro-inflammatory macrophages constitute the majority of monocyte-derived macrophages, which notably accumulate during corticotomy-mediated RAP.

### Corticotomy triggers ATF6 branch of the UPR in macrophages during OTM

To explore the crucial signal molecule regulating monocyte-derived macrophage polarization in corticotomy, we detected macrophage gene expression and function by our previous single-cell RNA-seq (GSE186185).^24^ Analysis revealed the enhanced effects of the ‘Endoplasmic Reticulum Unfolded Protein Response’ along with the elevated expression of UPR markers *Xbp1*, *Hspa5(Bip)* and *Ddit3(Chop)* in total and monocyte-derived macrophages during orthodontic alveolar bone remodeling (Fig. S3). Moreover, the increased co-localization of F4/80^+^BiP^+^GFP^−^ in the TM + CO group and the elevated proportion of BiP^+^ cells in GFP^−^ macrophages collectively indicated the potential triggering of UPR in monocyte-derived macrophages (Figs. 1f–h and S2c).

**Fig. 2:**
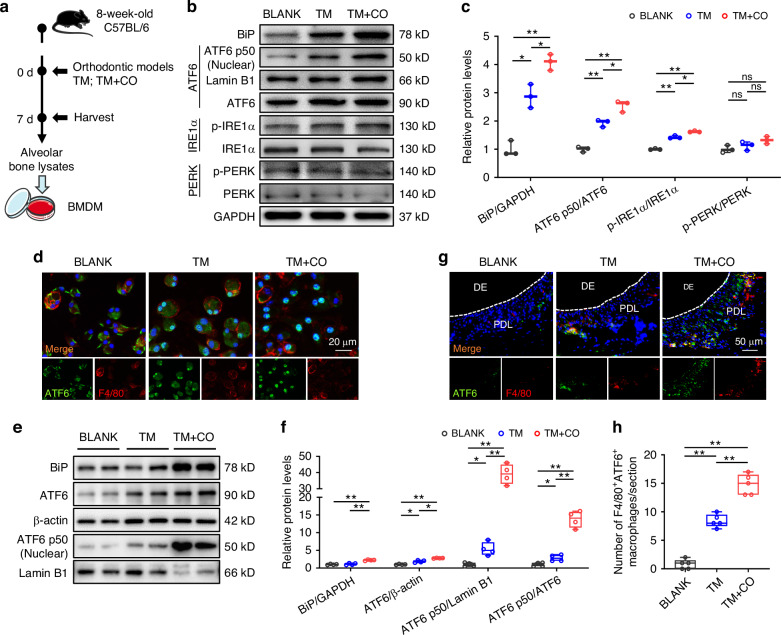
Corticotomy triggers ATF6 branch of the UPR in macrophages during OTM. **a** Experimental design: 8-week-old male C57B/L6 mice were randomly divided into BLANK, TM and TM + CO groups. After 7 days of OTM, the maxillae were harvested for alveolar bone lysates preparation. **b**, **c** Western blot analysis and quantification of ATF6 p50, ATF6, p-IRE1α, IRE1α, p-PERK, PERK and BiP protein expression in BMDMs with stimulation of alveolar bone lysates from Blank, TM and TM + CO mice. **d** Representative immunofluorescence staining images of ATF6^+^ (green) F4/80^+^ (red) cells with stimulation of alveolar bone lysates in vitro. Scale bars, 20 μm. **e**, **f** Western blot analysis and quantification of BiP, ATF6 and ATF6 p50 protein expression in murine periodontal tissues acquired 7 days after TM and TM + CO treatment. **g**, **h** Representative immunofluorescence staining images and quantification of ATF6^+^ (green) F4/80^+^ (red) cells in murine periodontal tissues acquired 7 days after TM and TM + CO treatment. Scale bars, 50 μm. Data represent mean ± SD. N = 3 or 4. *P < 0.05, **P < 0.01. ns no significance, TM tooth movement, TM + CO corticotomy-assisted orthodontic tooth movement

To elucidate the dynamics of UPR activation during corticotomy-mediated OTM acceleration, we mimic the microenvironment of OTM in vitro. Bone marrow-derived macrophages (BMDMs) were stimulated with fresh alveolar tissue lysates prepared from periodontal tissues surrounding the first molar 7 days post OTM modeling (Fig. 2a). Western blot and RT-PCR analyses revealed a significant activation of the UPR, especially ATF6 in the TM + CO group, with less activation of IRE1α and PERK (Figs. 2b, c and S4). The corticotomy-induced ATF6 activation in BMDMs was also confirmed by immunofluorescence staining (Fig. 2d).

We further detected the UPR signaling in alveolar bone tissues harvested from BLANK, TM, and TM + CO mice to verify the involvement of ATF6 branch during corticotomy-accelerated OTM. We found the ATF6 branch of the UPR significantly activated in the TM + CO group as evidenced by the increased expression of BiP, ATF6, and nuclear ATF6 p50 (Fig. 2e, f). Moreover, immunofluorescence staining demonstrated the increased co-localization of ATF6^+^F4/80^+^ in the TM + CO group (Fig. 2g, h). Collectively, these data suggest that corticotomy induces macrophage ATF6 activation during OTM.

### Macrophage ATF6 deficiency inhibits orthodontic bone remodeling in corticotomy through suppressing pro-inflammatory response

To evaluate the role of ATF6 in macrophages during orthodontic bone remodeling, we crossbreed ATF6^f/f^ mice with CX3CR1^CreERT2^ mice. We first observed the general bone phenotypes of tamoxifen-inducible macrophage-specific ATF6-knockout (ATF6^cKO^) mice. Results indicated that the specific deletion of ATF6 in macrophages showed no statistical differences in bone-related parameters and osteoclastogenesis (Figs. S5 and S6).

**Fig. 3:**
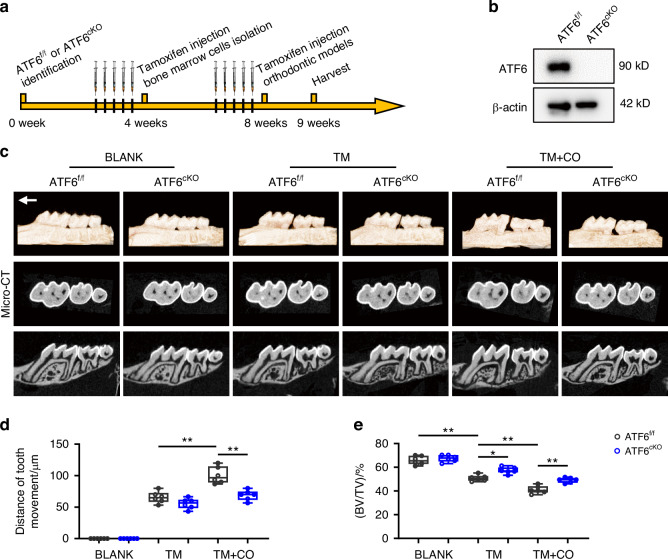
Macrophage ATF6 deficiency inhibits orthodontic bone remodeling in corticotomy. **a** Experimental design: ATF6^f/f^ and ATF6^cKO^ mice were intraperitoneally injected with tamoxifen (75 mg/kg) for 5 consecutive days. Orthodontic devices and corticotomy were applied to 8-week-old mice after 5 days of injections. After 7 days of OTM, the maxillae were harvested. Bone marrow cells were isolated from 4-week-old mice after tamoxifen injections. **b** Western blot analysis of ATF6 protein expression in BMDMs from ATF6^f/f^ or ATF6^cKO^ mice. **c** Representative 3D reconstruction images of the tooth movement distance after 7-day application of orthodontic force and corticotomy in ATF6^f/f^ or ATF6^cKO^ mice. **d**, **e** Quantitative parameters of micro-CT, including tooth movement distance (**d**) and bone volume relative to tissue volume (**e**) were shown. Data represent mean ± SD. N = 6. *P < 0.05, **P < 0.01. TM tooth movement, TM + CO corticotomy-assisted orthodontic tooth movement

Next, we performed TM or TM + CO surgery in ATF6^f/f^ and ATF6^cKO^ mice. Specific ablation of ATF6 in macrophages was accomplished via intraperitoneal injection of tamoxifen over 5 consecutive days prior to the establishment of TM and TM + CO models (Fig. 3a, b). We found that specific depletion of ATF6 in macrophages led to a significant reduced tooth movement distance in TM + CO group but did not affect the outcome of TM group (Fig. 3c, d). Furthermore, volumetric measurements affirmed that bone volume/tissue volume ratio (BV/TV) was diminished following corticotomy but showed a slight increase in ATF6^cKO^ mice compared to their ATF6^f/f^ littermates (Fig. 3e). In addition, the increased BV/TV in ATF6^cKO^ mice after TM + CO surgery was further validated by H&E staining (Fig. S7a, c). Moreover, TRAP staining demonstrated that ATF6 knockout reversed the rising of osteoclasts induced by corticotomy in periodontal tissues (Fig. S7b, d, e). Taken together, these findings underscore that specific knockout of ATF6 in macrophages significantly attenuates periodontal inflammatory bone remodeling following corticotomy.

**Fig. 4:**
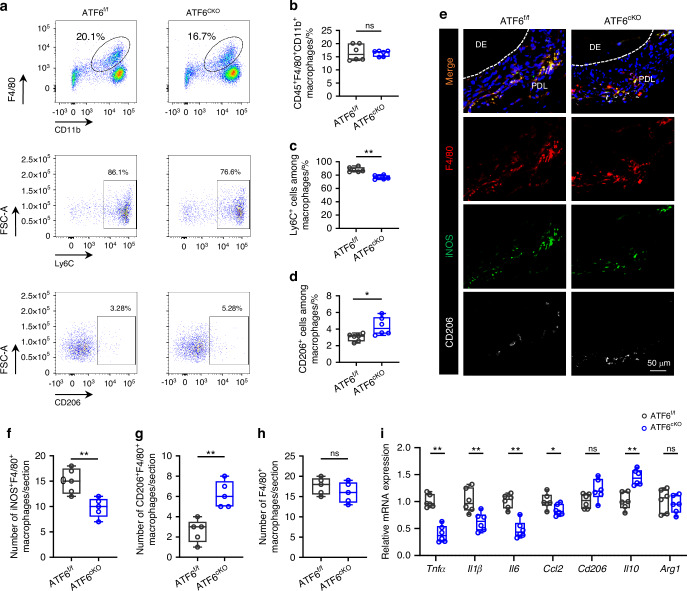
ATF6 deletion in macrophages suppresses pro-inflammatory response. **a**–**d** Gating strategies (**a**) and FACS analysis of macrophage ratio (**b**), Ly6C^+^ pro-inflammatory macrophage ratio (**c**), and CD206^+^ reparative macrophage ratio (**d**) in periodontal tissues after 7-day application of orthodontic force and corticotomy in ATF6^f/f^ or ATF6^cKO^ mice. **e** Representative immunofluorescence staining of iNOS^+^ (green) F4/80^+^ (red) cells and CD206^+^ (white) F4/80^+^ (red) cells in periodontal tissues after 7-day application of orthodontic force and corticotomy in ATF6^f/f^ or ATF6^cKO^ mice. Scale bars, 50 μm. **f**–**h** Quantification of iNOS^+^ (green) F4/80^+^ (red) cells (**f**), CD206^+^ (white) F4/80^+^ (red) cells (**g**), and F4/80^+^ (red) cells (**h**) in periodontal tissues from ATF6^f/f^ or ATF6^cKO^ mice treated with orthodontic force and corticotomy. **i** RT-PCR analysis of *Tnfα*, *Il1β*, *Il6*, *Ccl2*, *Cd206*, *Il10* and *Arg1* mRNA expression in periodontal tissues of ATF6^f/f^ or ATF6^cKO^ mice treated with orthodontic force and corticotomy. Data represent mean ± SD. N = 5 or 6. *P < 0.05, **P < 0.01. ns no significance, TM tooth movement, TM + CO corticotomy-assisted orthodontic tooth movement

To substantiate the influence of ATF6 on macrophage responses to corticotomy, flow cytometry was used to analyze macrophage polarization in vivo (Fig. 4a). The proportion of CD45^+^F4/80^+^CD11b^+^ macrophages did not exhibit statistically significant differences between these two groups (Fig. 4b). The ensuing data indicated a decrease in the percentage of pro-inflammatory CD45^+^F4/80^+^CD11b^+^Ly6C^+^ macrophages in periodontal tissues of ATF6^cKO^ mice (Fig. 4c), while the percentage of restorative CD45^+^F4/80^+^CD11b^+^CD206^+^ macrophages exhibited a slight increase (Fig. 4d). Immunofluorescence staining echoed these trends, as the iNOS^+^F4/80^+^ cells waned in periodontal tissues from ATF6^cKO^ mice with TM + CO model (Fig. 4e, f), while the CD206^+^F4/80^+^ cells was slightly elevated compared to ATF6^f/f^ mice (Fig. 4e, g). The overall macrophage count remained consistent (Fig. 4h). Moreover, we also observed a significant attenuation of pro-inflammatory marker genes *Tnfα*, *Il1β*, *Il6* and *Ccl2* in the periodontal tissues of ATF6^cKO^ mice compared to their control littermates (Fig. 4i). Collectively, these findings suggest that the deficiency of ATF6 in macrophages impedes the function of pro-inflammatory macrophages, consequently reversing the acceleration of tooth movement in response to corticotomy.

### Macrophage-specific ATF6 overexpression accelerates corticotomy-assisted OTM

To further confirm the role of macrophage ATF6 in alveolar bone remodeling during corticotomy, we generated a macrophage specific ATF6 overexpression lentivirus guided by the SP-C1 promoter (Fig. S8a, b). Mice received tail vein injection of either the ATF6 overexpression or control lentivirus. CD45^+^F4/80^+^CD11b^+^ macrophages were sorted out from mouse periodontal tissues via flow cytometry post-lentivirus injection and subjected to RT-PCR analysis. The heightened macrophage count and the amplified expression of macrophage ATF6 corroborated the successful lentivirus manipulation (Fig. S8c, d).

**Fig. 5:**
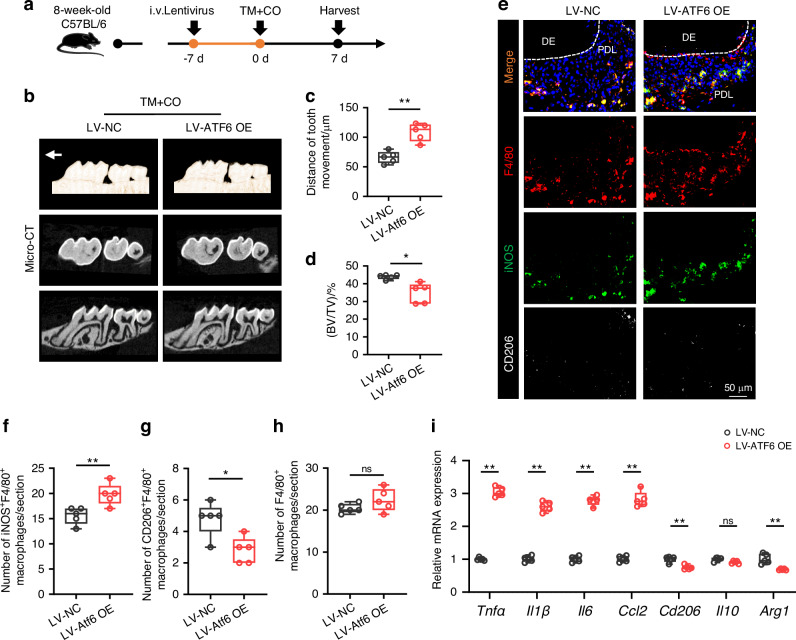
Macrophage-specific ATF6 overexpression accelerates corticotomy-assisted OTM. **a** The detailed experimental design for overexpression of macrophage ATF6 by prior administration of lentivirus into TM + CO mice. Mice were sacrificed 7 days after orthodontic treatment. **b** Representative 3D reconstruction images of the tooth movement distance of ATF6 overexpressed TM + CO mice. **c**, **d** Quantification of tooth movement distance (**c**) and bone volume relative to tissue volume (**d**) were shown. **e** Representative immunofluorescence staining of iNOS^+^ (green) F4/80^+^ (red) cells and CD206^+^ (white) F4/80^+^ (red) cells in periodontal tissues from TM + CO mice with overexpression lentivirus administration. Scale bars, 50 μm. **f**–**h** Quantification of iNOS^+^ (green) F4/80^+^ (red) cells (**f**), CD206^+^ (white) F4/80^+^ (red) cells (**g**), and F4/80^+^ (red) cells (**h**) in periodontal tissues was shown. **i** RT-PCR analysis of *Tnfα*, *Il1β*, *Il6*, *Ccl2*, *Cd206*, *Il10* and *Arg1* mRNA expression in periodontal tissues from TM + CO mice injected with overexpression lentivirus or negative control. Data represent mean ± SD. N = 5. *P < 0.05, **P < 0.01. ns, no significance. TM + CO corticotomy-assisted orthodontic tooth movement, LV-ATF6 OE ATF6-gene-overexpressed lentivirus, LV-NC scrambled lentivirus served as a negative control

Next, we established the TM + CO models 7 days after lentivirus injection (Fig. 5a). In vivo experiment revealed that the overexpression of ATF6 in macrophages amplified orthodontic tooth movement acceleration induced by corticotomy (Fig. 5b, c) and attenuated bone volume (BV/TV) (Fig. 5d). Enhanced osteoclast activity was discerned in ATF6 overexpression models compared to controls, as confirmed by H&E and TRAP staining (Fig. S8e–g). Notably, immunofluorescence staining verified an increase in the number of iNOS^+^F4/80^+^ pro-inflammatory macrophages (Fig. 5e, f), whereas the count of CD206^+^F4/80^+^ restorative macrophages was diminished (Fig. 5e, g). However, the total macrophage count remained unchanged (Fig. 5h). Further, RT-PCR analysis of local alveolar bone indicated elevated expression of pro-inflammatory markers at mRNA levels (Fig. 5i). Taken together, these findings suggest that ATF6 in macrophages may potentiate the acceleration of OTM by augmenting pro-inflammatory macrophage activity.

### ATF6 is required for macrophage pro-inflammatory signals in vitro

**Fig. 6:**
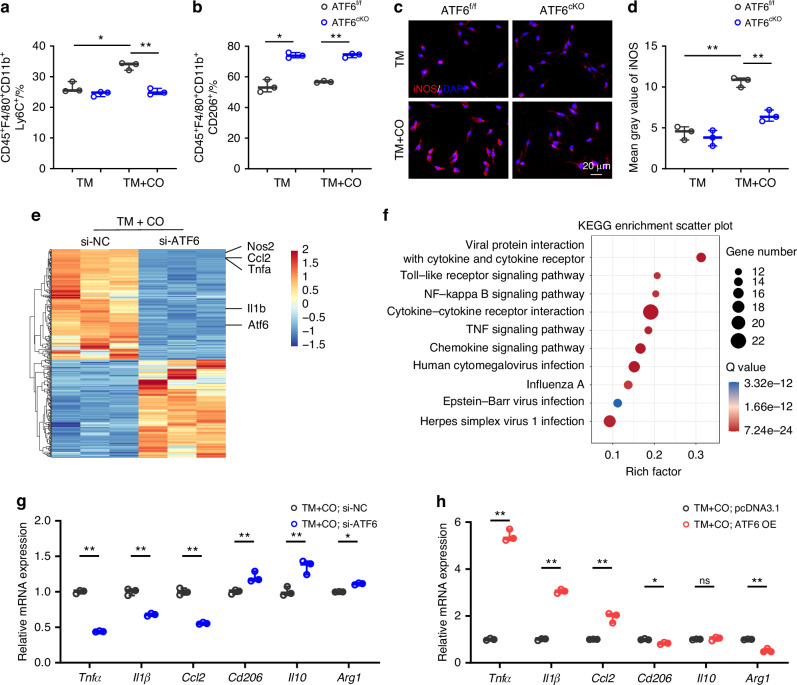
ATF6 is required for macrophage pro-inflammatory signals in vitro. **a**, **b** Flow cytometric analysis of Ly6C^+^ pro-inflammatory macrophage ratio (**a**) and CD206^+^ reparative macrophage ratio (**b**) in BMDMs from ATF6^f/f^ or ATF6^cKO^ mice upon stimulation of alveolar bone lysates from TM or TM + CO mice. **c**, **d** Representative immunofluorescence staining and quantification of iNOS^+^ (red) cells in BMDMs from ATF6^f/f^ or ATF6^cKO^ mice under stimulation of alveolar bone lysates from TM or TM + CO mice. Scale bars, 20 μm. **e** Heatmap of DEGs of BMDMs infected with ATF6-siRNA or negative control under stimulation of alveolar bone lysates from TM + CO mice. Blue and red colors represent low and high expression values, respectively. **f** Representative downregulated KEGG pathway analysis influenced by ATF6 knockdown. **g**, **h** RT-PCR analysis of pro-inflammatory markers *Tnfα*, *Il1β* and *Ccl2* and anti-inflammatory markers *Cd206*, *Il10* and *Arg1* mRNA expression in BMDMs infected with ATF6-siRNA or negative control (**g**) and ATF6 overexpress plasma or negative control plasma (**h**) under stimulation of alveolar bone lysates from TM + CO mice. Data represent mean ± SD. N = 3. *P < 0.05, **P < 0.01. ns no significance, TM tooth movement, TM + CO corticotomy-assisted orthodontic tooth movement, ATF6-siRNA small interference ATF6, NC negative control

To understand the impact of ATF6 in macrophage inflammatory function, we cultured BMDMs from ATF6^f/f^ and ATF6^cKO^ mice stimulated with alveolar bone lysates. FACS analysis revealed that the heightened percentages of CD45^+^F4/80^+^CD11b^+^Ly6C^+^ cells in the TM + CO group compared to the TM group were attenuated by ATF6 knockout (Figs. 6a and S9a). Moreover, the proportions of CD45^+^F4/80^+^CD11b^+^CD206^+^ cells were elevated in BMDMs derived from ATF6^cKO^ mice relative to those from ATF6^f/f^ mice, although no significant discrepancy was discernible between the TM and TM + CO groups (Figs. 6b and S9b). Immunofluorescence staining yielded a similar outcome, revealing that ATF6 knockout entirely blocked the increase of iNOS expression with the conditioned medium from the TM + CO group (Fig. 6c, d).

To dissect the mechanism by which ATF6 modulates macrophage inflammatory signals, RNA-sequencing (RNA-seq) was performed on BMDMs transfected with negative control siRNA (si-NC) and ATF6 siRNA (si-ATF6) upon TM + CO stimulation. Using the Q value (Q value < 0.05) and the expression fold change (Fold change ≥2 or ≤0.5) as criteria, we identified 175 downregulated and 155 upregulated genes in ATF6-siRNA BMDMs compared to negative control BMDMs under conditioned medium treatment from the TM + CO group. Notably, several pro-inflammatory marker genes including *Tnfα*, *Ccl2*, *Nos2*, and *Il1β* were among the top downregulated genes (Fig. 6e). Further KEGG analysis of the significantly altered genes indicated that the downregulated pathways in ATF6 knockdown BMDMs were associated with inflammation and immune response in macrophages, especially NF-κB and TNF signaling pathway (Fig. 6f). GSEA confirmed a significant downregulation of the NF-κB signaling pathway and the TNF signaling pathway due to ATF6 deficiency (Fig. S9c, d). Concordant with the RNA-seq data, the expression of pro-inflammatory marker genes *Tnfα*, *Il1β*, and *Ccl2* significantly decreased, while the anti-inflammatory marker genes *Cd206*, *Il10*, and *Arg1* slightly increased when ATF6 was knocked down in BMDMs (Fig. 6g). Finally, overexpression of ATF6 upregulated the expression of pro-inflammatory marker genes and reduced the expression of anti-inflammatory marker genes in the context of TM + CO stimulation (Fig. 6h). In summary, these data, in alignment with the preceding findings, unequivocally emphasize a pivotal role of ATF6 in governing macrophage pro-inflammatory signals in vitro.

### ATF6 promotes macrophage-mediated OTM through activating Tnfα transcription

**Fig. 7:**
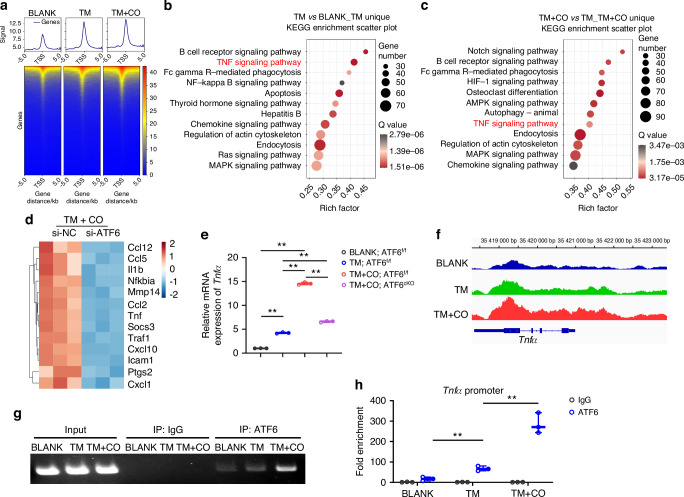
ATF6 activates Tnfα transcription during corticotomy. **a** Line graphs and heat maps of ATF6 occupancy on the promoter region (TSS ± 5.0) kb, ranked according to ATF6 signal density in BMDMs from Blank, TM and TM + CO groups. **b**, **c** KEGG analysis of unique peaks of the top 12 enriched pathways in BMDMs related to inflammatory and immune responses in TM *vs* Blank group (**b**) and TM + CO *vs* TM group (**c**). **d** Heat maps of genes associated with TNF signaling pathway based on the results of RNA-seq. Blue and red colors represent low and high expression values, respectively. **e** RT-PCR analysis of Tnfα expression in BMDMs from ATF6^f/f^ or ATF6^cKO^ mice with stimulation of alveolar bone lysates from Blank, TM or TM + CO mice. **f** IGV snapshot of peak values of ATF6 p50 binding on *Tnfα* gene in BMDMs from BLANK, TM and TM + CO groups. **g**, **h** ChIP assay verification for the binding of ATF6 p50 to *Tnfα* promoter in BMDMs treated with tissue suspension from BLANK, TM and TM + CO group. Data represent mean ± SD. N = 3. **P < 0.01. TM tooth movement, TM + CO corticotomy-assisted orthodontic tooth movement

To further investigate the mechanism by which ATF6 influences macrophage pro-inflammatory function, we sought to identify candidate target inflammatory cytokines of ATF6 p50 in BMDMs. To this end, BMDMs were stimulated with alveolar bone lysates from BLANK, TM, and TM + CO models for 3 h. Cleavage Under Targets & Tagmentation (CUT&Tag) assay showed the recruitment of ATF6 to new sites in response to the stimulation of inflammation microenvironments (Fig. 7a) and significant enrichment of the TNF signaling pathway in the TM group in comparison with the BLANK group (Fig. 7b). We also yielded a similar outcome, demonstrating enrichment of the TNF signaling pathway in the TM + CO group relative to the TM group (Fig. 7c). Both RNA-Seq and CUT&Tag data suggested a positive correlation between ATF6 and the expression of *Tnfα*, the most dominant macrophage secreted cytokine modulating OTM^25^ (Fig. 7d). RT-PCR analysis further verified the elevation of *Tnfα* expression observed in BMDMs from the TM + CO group was significantly reversed in the ATF6^cKO^ group (Fig. 7e). This result suggests that the specific knockout of ATF6 can suppress the local inflammatory microenvironment by downregulating TNFα expression. Next, we employed the Integrative Genomics Viewer (IGV) software to visualize the highest peak values of ATF6 p50 binding on the *Tnfα* promoter (ranging from −2 000 to 0 bp upstream of the transcription start site) (Fig. 7f). ChIP assay further demonstrated that ATF6 p50 could directly bind to the promoter region of *Tnfα* (Fig. 7g, h). Although *Il1β* was also changed after ATF6 knockout, our findings suggested that ATF6 did not mediate its transcription, indicating it might be the adjoint reaction of the TNF signaling activation (Fig. S10).

**Fig. 8:**
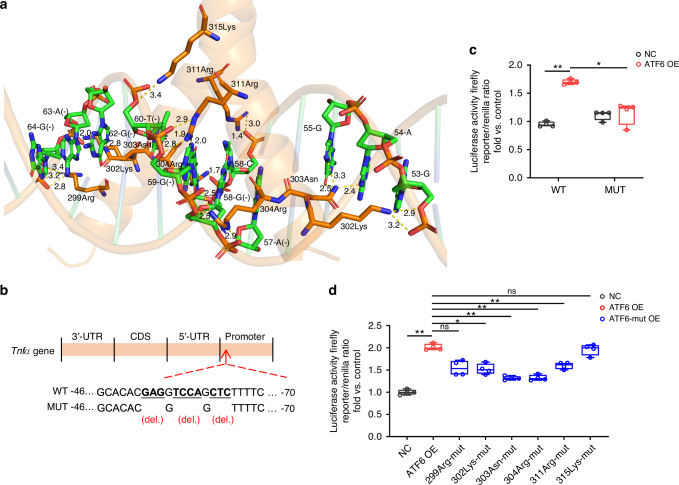
ATF6 p50 binds to specific sites of the Tnfα promoter. **a** Bioinformatics analysis of the specific binding locations of ATF6 p50 to *Tnfα* promoter. (Yellow dashed lines indicate hydrogen bonding interactions that occur between amino acid residues of proteins and DNA bases, and numbers on dashed lines indicate bond lengths). **b** Diagram of the binding locations of ATF6 p50 in the *Tnfα* 5′-flanking region, their wild-type sequences (WT), and the mutation to those sequences (MUT) (mutation site: red). **c** Luciferase reporter assay of 293 T cells after transfecting the WT or MUT *Tnfα* promoter. **d** Luciferase reporter assay of 293 T cells co-transfected with WT-*Tnfα* promoter-Luc-and ATF6 or ATF6-mut plasmids. Data represent mean ± SD. N = 4. *P < 0.05, **P < 0.01. ns no significance, del. deletion mutation

Furthermore, the 3D structure of the ATF6 p50 interacted with the promoter region of *Tnfα* was conducted computationally, illustrating a functional binding contact between the critical amino acids (299Arg, 302Lys, 303Asn, 304Arg, 311Arg and 315Lys) in basic motif of bZIP domain of ATF6 and promoter region of the *Tnfα* gene (Fig. 8a). Following the protein-DNA interaction prediction, luciferase reporter plasmids containing about 2000 bp of the wild-type and mutant *Tnfα* promoters were constructed (Fig. 8b). The dual-luciferase reporter assay showed that the transcriptional activity of the *Tnfα* promoter was significantly enhanced by ATF6 and diminished by mutations of the ATF6 binding region in the *Tnfα* promoter (Fig. 8c). Furthermore, the transcription activity of ATF6 against *Tnfα* was notably decreased after mutation of 302Lys, 303Asn, 304Arg and 311Arg in ATF6, which are the key interaction residues of ATF6-*Tnfα* transcription promotion (Fig. 8d). These results suggest that ATF6 directly binds to the *Tnfα* promoter and augments its transcription, thereby offering a potential mechanism for the influence of macrophage ATF6 on mediating critical pro-OTM cytokine, TNFα.

### TNFα rescues OTM inhibition induced by macrophage ATF6 knockout

**Fig. 9:**
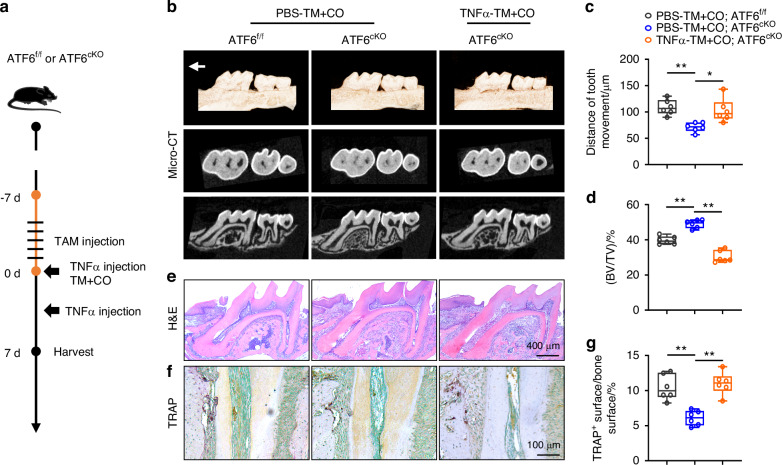
TNFα rescues macrophage ATF6 knockout induced OTM inhibition. **a** Experimental design: ATF6^f/f^ and ATF6^cKO^ mice were intraperitoneally injected with tamoxifen (75 mg/kg) for 5 consecutive days. After 5 days, 8-week-old mice were periodontally injected with 10 μl TNFα (20 μg/mL) and applied with orthodontic devices and corticotomy. Three days later, the TNFα injections were repeated. After 7 days of OTM, the maxillae were harvested. **b** Representative 3D reconstruction images of the tooth movement distance after 7-day application of orthodontic force and corticotomy in ATF6^f/f^ or ATF6^cKO^ mice with or without TNFα periodontal injection. **c**, **d** Quantification of the tooth movement distance (**c**) and bone volume relative to tissue volume (**d**) were shown. **e**, **f** Representative H&E staining (**e**) and TRAP staining (**f**) images of murine first molar after 7-day application of orthodontic force and corticotomy in ATF6^f/f^ or ATF6^cKO^ mice with or without TNFα periodontal injection. Scale bars of H&E staining images, 400 μm. Scale bars of TRAP staining images, 100 μm. **g** Quantification of surface of osteoclasts relative to the bone surface was shown. Data represent mean ± SD. N = 6. *P < 0.05, **P < 0.01. TM + CO corticotomy-assisted orthodontic tooth movement

Finally, to assess the dependence of ATF6-TNFα axis in promoting alveolar bone resorption and facilitating orthodontic tooth movement (OTM), TM + CO models were applied to ATF6^f/f^ and ATF6^cKO^ mice with or without TNFα injection (Fig. 9a). Micro-CT scanning and three‐dimensional reconstruction revealed that TNFα injection significantly restored the reduced OTM distance and reversed the increased bone volume (BV/TV) caused by macrophage ATF6 knockout (Fig. 9b–d). H&E and TRAP staining showed that TNFα injection counteracted the inhibitory effect of macrophage ATF6 on osteoclast activity during corticotomy (Fig. 9e–g). These data supported the notion that TNFα serves as a crucial pro-OTM remodeling factor downstream of ATF6 in response to corticotomy. These findings provide evidence for ATF6 or TNFα being potential clinical targets in accelerated orthodontic tooth movement and alveolar bone remodeling.

## Discussion

**Fig. 10:**
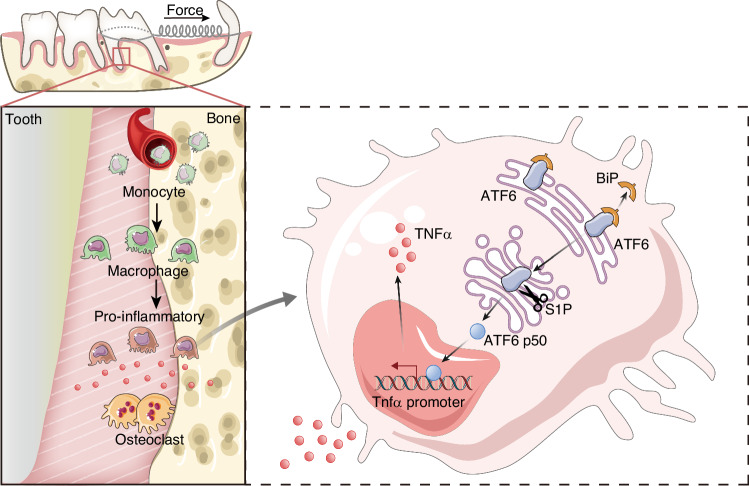
Graphic abstract of this study

Among the attempts of innovative techniques to accelerate OTM, corticotomy appears to show strong effect by activating RAP.^3,26,27^ Here, we showed that monocyte-derived pro-inflammatory macrophages serve as a critical immune modulator during corticotomy-induced RAP. More importantly, macrophage ATF6 deficiency inhibits pro-inflammatory macrophage polarization and blocks the corticotomy-induced acceleration of OTM in a murine model, highlighting the importance of macrophage ATF6 in alveolar bone remodeling. Further, we provided evidence that ATF6 may play an essential role in pro-inflammatory macrophage polarization through activating the *Tnfα* transcription, which amplifies the effect of TNFα to promote OTM (Fig. 10).

RAP is a concept that describes how an intentional surgical injury to bone starts a cascade of physiologic events, leading to an increased rate of bone remodeling with concomitant demineralization and new bone formation in the region of the bone injury.^28–30^ It has been reported as a vivid display of osteoimmunology, an emerging field that describes the intimate relationship between bone biology and the immune system.^31^ Macrophages, as a key regulator of the immune response, have been shown to actively participate in the bone remodeling processes.^24,32^ Notwithstanding the phenotypic switch of macrophages during orthodontic bone remodeling,^9,33^ the origin of macrophages remains unexplored. Monocyte-derived macrophages contribute to tissue integrity as well as to innate and adaptive immune defense, while tissue-resident macrophages originate from early yolk sac progenitors and contribute to tissue metabolism, serving as healers of infection and tissue damage.^34^ Our study employed lineage tracing to uncover the specific roles of tissue-resident and monocyte-derived macrophages in corticotomy and highlight the dominant role of monocyte-derived macrophages in the acceleration of OTM through their orchestration of inflammation.

The ER stress sensor ATF6 has been implicated in promoting pro-inflammatory signaling events during various inflammatory diseases.^18,35–37^ Macrophage ATF6 has been revealed to enhance TLR4 response and elevate the ER stress-mediated pro-inflammatory signal via NF-κB pathway.^18,37^ It has also been proposed that ATF6 upregulates AIFM2 and promotes p53-induced apoptosis,^36^ and engagement of other pro-inflammatory signaling events such as the activation of p38MAPK and JNK have also been reported during ER stress.^35,38^ However, its role in bone remodeling and OTM remains poorly understood. Our study revealed that corticotomy triggered changes in the alveolar bone microenvironment, leading to ER stress and subsequent activation of ATF6. By investigating the contribution of ATF6 in corticotomy-acceletated OTM, we aimed to shed light on its involvement in inflammatory bone remodeling processes.

ATF6 operates as a basic-leucine zipper (bZIP) transcription factor that binds to the consensus ER stress response element (ERSE), regulating the transcription of genes involved in maintaining ER homeostasis, such as ER chaperones and ER-associated protein degradation (ERAD) components.^39,40^ The bZIP domain contains a basic region mediating sequence-specific DNA-binding followed by a leucine zipper region required for dimerisation.^41^ Interestingly, the unique effects of stimulus influencing ATF6 binding sites and transcriptionally activating different signaling pathways have been uncovered recently.^42^ Our study clarified the amino acid binding site by bioinformatic prediction and experimental verification, underscoring the broad genomic binding capability of ATF6 across various cell types and its potential to influence gene expression patterns.

One of the significant findings of our study is the identification of ATF6 as a critical regulator of *Tnfα* transcription, which may offer novel inflammatory response elements as ATF6 binding sites. TNFα induces the acceleration of tooth movement and also plays an important role in the pathogenesis of osteolysis in inflammatory diseases.^43,44^ Although several lines of evidence have demonstrated that ATF6 is relevant to TNFα induction, underlying molecular mechanisms are lacking.^18,35^ Considering this correlation, the specific binding locations of ATF6 p50 to *Tnfα* promoter were predicated upon bioinformatics analysis and confirmed by site mutation. We showed that ATF6 p50 binds to the promoter region of *Tnfα* and enhances its transcriptional activity, thereby enlarging the effect of TNFα in accelerating OTM. The newly identified binding site, rather than ERSE, may be a novel binding element for ATF6 in the inflammatory microenvironment.

ATF6 plays a critical role in modulating alveolar bone remodeling during corticotomy. However, our study showed that ATF6 deletion had no significant effect on the general bone phenotypes of ATF6^cKO^ mice and did not directly influence osteoclast differentiation and function in vitro. Our findings suggest that ATF6-driven *Tnfα* transcription modulates the inflammatory microenvironment around the periodontal tissues. TNFα, a pivotal pro-inflammatory cytokine involved in bone resorption, promotes osteoclast-related gene expression and synergizes with nuclear factor kappa-B ligand (RANKL) signaling, which subsequently stimulates osteoclastogenesis under inflammatory conditions.^45–47^ This process ultimately accelerates the bone remodeling necessary for effective tooth movement. It is suggested that ATF6 does not directly affect osteoclast differentiation but rather modulates their activity through an inflammatory signaling cascade, underscoring its role in regulating bone resorption during orthodontic tooth movement.

Studies have demonstrated that alveolar bone density initially decreases following corticotomy but increases during the late stage.^48,49^ The therapeutic osteopenic state of RAP is known to be physiologic, intentional, temporary, and fully reversible, which differs from the pathological state of osteoporosis. Our study indicates that corticotomy activates ATF6 in macrophages during the initial phase of tooth movement, promoting osteoclastogenesis and bone resorption through the activation of the ATF6-TNFα axis. Notably, osteoclasts secrete active substances such as TGF-β and Wnt10b,^50,51^ which promote osteoblast differentiation and maintain alveolar bone regeneration.^52^ Additionally, excessive activation of ATF6 leads to apoptosis of pro-inflammatory macrophages, which helps regulate the intensity and duration of the inflammatory response, thereby restricting the destructive potential of TNFα exposure.^13,53^ Future investigations into short-term modulation of ATF6 expression using small molecular compounds may help reduce adverse effects associated with prolonged TNFα stimulation while enhancing the therapeutic efficiency. Macrophages are also known to display a remarkable plasticity. After the acute phase, macrophages transform into restorative ones and play a role in tissue repair in the later stage of corticotomy.^8^ Therefore, regulating macrophage phenotypes is a powerful approach for controlling inflammation and influencing orthodontic tooth movement. By clarifying that TNFα is the key downstream effector of ATF6 in the context of corticotomy, we were able to propose a more targeted and precise regulatory strategy aimed at accelerating orthodontic tooth movement and maintaining the long-term alveolar bone homeostasis.

In summary, our study provides novel insights into the molecular mechanisms underlying RAP. We identify a vital role of ATF6-mediated UPR in regulating macrophage functional transition through *Tnfα* transcription during corticotomy. By modulating ATF6 activity, it may be possible to facilitate alveolar bone remodeling and reduce the tissue damage caused by corticotomy. As pharmacologic ATF6 activators continue to advance, future research could explore the use of exosomes or novel biomaterials to selectively enhance ATF6 activation in bone tissues.^54–56^ Targeting the ATF6-TNFα axis may pave the way for potential non-invasive therapeutic interventions aimed at improving orthodontic treatment outcomes.

## Materials and methods

### Animals

Both wild type and CX3CR1^CreERT2^; R26^GFP^ mice in C57BL/6 background (Gempharmatech Co., Ltd) were used in our study. Mononuclear phagocytes targeted ATF6 receptor null mice (referred to as ATF6^cKO^ mice) were generated on the C57BL/6 genetic background (ATF6^f/f^; CX3CR1^CreERT2^) by crossing CX3CR1^CreERT2^ mice (C57BL/6 background, Jackson Laboratory) into ATF6^f/f^ mice.

All mice were maintained in the SPF Medical Experimental Animal Center of Nanjing Medical University, China. All aspects of the animal care and experimental protocols were approved by the Committee of Nanjing Medical University for Animal Resources (approval ID: IACUC‐1601118).

### Drug administration

Tamoxifen (T5648; Sigma) was dissolved in corn oil (C7030; Solarbio) to get a 20 mg/mL solution. To induce Cre-mediated gene recombination, the transgenic mice were intraperitoneally injected with tamoxifen (75 mg/kg) for 5 consecutive days.

The ATF6^cKO^ mice were randomly divided into 2 groups after tamoxifen injection. 10 μL of 20 μg/mL TNFα solution (315-01 A; PeproTech) was injected to the maxillary first molar periodontal area using a graded Hamilton syringe (34-G needle), and the vehicle group was injected with PBS every three days.

### Application of orthodontic devices and corticotomy

Certain modification was made based on previous studies.^8,57^ For the orthodontic tooth movement (TM) model, nickel-titanium coil springs with a diameter of 0.008 inches were ligated between the left maxillary first molar and maxillary incisors of mice to induce mesial movement of molars. To construct a model of corticotomy-assisted orthodontic tooth movement (TM + CO), two vertical corticotomies with a diameter of 1 mm and a depth of 2 to 3 mm were performed on the mesial and distal side of mice first molar. Micro-CT scanning was performed after modeling to ensure consistency of the model. The orthodontic devices were checked every day. The mice were allowed free access to water and soft food after installation to relieve discomfort. Animals were randomly assigned to the TM and TM + CO groups, and sacrificed 0, 1, 3, 5, 7 and 14 days after force application.

### Micro-computed tomography scanning and analysis

Maxillae were collected from each group and scanned using a micro-computed tomography (micro‐CT) machine (vivaCT80, Switzerland) with a standard acquisition protocol (55 kV, 72 μA, and 15.6 μm voxel size). A single‐blinded rater renamed the micro‐CT data with randomly generated codes, and imported the files into InVivoDental for 3D reconstruction. After reorientation, orthodontic tooth movement distance was determined by the most mesial point of the second molar crown and the most distal point of the first molar crown. The files were also imported into Mimics software (Materialise) for 3D reconstruction and longitudinal sections. Volumetric measurements were then performed in CTAn v1.15.4.0 according to the previous report.^58^ Shortly, the alveolar bone between the mesiobuccal root and distobuccal root of the first maxillary molar on the sagittal view was defined as the region of interest (ROI). Fifteen continuous images beginning from this ROI were used for 3-dimensional reconstruction and analysis (Fig. S11a).

### Histological staining

Maxillae collected from sacrificed mice were fixed in 4% paraformaldehyde solution (PFA) for 48 h and decalcified in 14% ethylenediaminetetraacetic acid (EDTA) for 4 weeks. The dehydrated maxillae were embedded in paraffin and cut into 4 μm slices from the middle part (between the double black bars) (Fig. S11b). Then, the paraffin sections were evaluated by haematoxylin and eosin (H&E) staining (Hematoxylin and Eosin Staining Kit, Beyotime) and tartrate-resistant acid phosphatase (TRAP) staining (TRAP Kit 387 A, Sigma-Aldrich). Osteoclast activity was identified by TRAP-positive signs.

### Tissue collection and lysates preparation

Maxillae were removed and dissected free of all soft tissues. After the extraction of first molars, the alveolar bone block of 5 mice in each group was collected in 5 mL Alpha-modified Minimum Essential Medium (α-MEM) with 1% fetal bovine serum (FBS) (Fig. S11c, d). As previously reported, the alveolar bone lysates were prepared with Medimachine (BD Biosciences) and filtered through a 0.22-μm filter (Millipore, MA, USA) to ensure sterility.^8,59^ To minimize sampling error and bias, these processes were performed by a single operator.

### Immunofluorescence staining

For immunofluorescence staining, the treated maxillae were embedded in Tissue-Tek O.C.T. compound, and cut into 8 μm of each slice for frozen sections. The sections were stained overnight with Rat anti‐F4/80 (1:100, ab16911; Abcam), Rabbit anti-BiP (1:100, 3177; Cell Signaling Technology), Rabbit anti-ATF6 (1:100, ab37149; Abcam) Rabbit anti-iNOS (1:100, ab15323; Abcam) or Goat anti-CD206 (1:100, AF2535; R&D system) at 4 °C. Sections were then incubated with Goat anti-Rat 594 (1:100, A0507; Beyotime), Goat anti-Rabbit 488 (1:100, SA00013-2; ProteinTech), Goat anti-Rabbit 649 (1:100, A23620; Abbkine) and Donkey anti-Goat 647 (1:500, ab150135; Abcam) at room temperature for 45 min. The frozen sections were mounted with Mounting Medium containing DAPI (ZF0812; VECTASHIELD).

In vitro cells were fixed with 4% PFA for 20 min at room temperature, permeabilized with 0.2% Triton X-100, and blocked with 10% normal goat serum for 30 min. The primary antibody used were against Rat anti‐F4/80 (1:100, ab16911; Abcam), Rabbit anti-ATF6 (1:100, ab37149; Abcam) or Rabbit anti-iNOS (1:100, ab15323; Abcam). On the next day, the samples were washed and incubated with the same secondary antibody as that used for frozen tissue sections for 1 h at room temperature. All images were captured using a fluorescence microscope (Carl Zeiss) and the representative images were selected randomly from each group.

### Flow cytometry and FACS gating strategies

Tissues were minced with gentleMACS Dissociator (gentleMACS) in ice‐cold PBS with 1% FBS, and filtered through a 200‐mesh nylongrid. For flow cytometric sorting, cells were harvested by centrifugation and then incubated with fluorochrome-conjugated antibodies against Zombie (1:100, 423113; BioLegend), CD45 (1:100, 553079; BD Biosciences), F4/80 (1:100, 123135; BioLegend) and CD11b (1:100, 17-0112-82; eBioscience), Ly6C (1:100, 128025; BioLegend), CD206 (1:100, 12-2061-82; eBioscience), BiP (1:50, 14464S; Cell Signaling Technology). Flow cytometry was performed using FACSAria II (BD Biosciences).

Mouse macrophage: CD45^+^F4/80^+^CD11b^+^; pro-inflammatory macrophage: CD45^+^F4/80^+^CD11b^+^Ly6C^+^; restorative macrophage: CD45^+^F4/80^+^CD11b^+^CD206^+^; monocyte-derived macrophage: GFP^−^CD45^+^CD11b^+^F4/80^+^; tissue-resident macrophage: GFP^+^CD45^+^CD11b^+^F4/80^+^.

### Lentivirus construction, infection, and injection

The FLAG-tagged mouse ATF6 (NM_001081304) with SP-C1 promoter was subcloned into the pLVX-SP-FLAG-P2A-mCherry vector (Supplemental Table I). Plasmids were purchased from Viraltherapy Technologies Co. (Wuhan, China) and packaged into lentivirus particles using HEK 293 T cells. To achieve a sustained overexpression effect in mice, lentivirus (1 × 10^8^ GC/mL, 100 μL) was injected into the male mice via tail vein. The mice were applied with orthodontic devices and corticotomy 7 days after injection. Lentivirus transduction efficiency in BMDMs was determined by western blot analysis.

### Isolation, culture, and treatment of bone marrow‐derived macrophages

Bone marrow cells were flushed out from the bone marrow cavity of femurs and tibias from 4 to 5 weeks male C57BL/6 mice using α-MEM with 2% foetal bovine serum (FBS). After lysis of red blood cells, these cells were cultured in α-MEM containing 10% FBS, supplemented with 1% penicillin/streptomycin (P/S), and 25 ng/mL M‐CSF at 37°C with 5% CO_2_ for 5 days to generate BMDMs. On day 5, BMDMs were treated with sterile tissue suspension for 6 h before protein detection and 3 h before mRNA detection.

### RNA extraction and real‐time polymerase chain reactions (RT-PCR) analysis

Total mRNA was extracted from murine alveolar bone tissues or BMDMs with TRIzol Reagent (15596026; Invitrogen). cDNAs were reversely transcribed from 1 μg of total RNA in a volumn of 10 μL with the PrimeScript RT Master Mix (Q711-02; Vazyme) and subjected for Quantitative real‐time PCR on an ABI QuantStudio7 (Applied Biosystems). The relative expression level for each mRNA was calculated using the 2^−ΔΔCt^ methods. The sequences of the forward and reverse primers are listed in Supplemental Table II.

### Cell lysis and western blot analysis

Cellular proteins were extracted with ice cold RIPA buffer and quantitated by BCA protein assay kit (P0012; Beyotime). Proteins (20 μg) were separated by 10% SDS-PAGE and transferred onto polyvinylidene diflouride (PVDF) membrane. After being blocked in 5% skim milk solution at room temperature, membranes were incubated with primary antibodies at 4 °C overnight. Western blot primary antibodies include: BiP (1:1 000, 3177; Cell Signaling Technology), ATF6 (1:1 000, NBP1-40256; Novus Biologicals) for cytoplasm protein, ATF6 (1:1 000, ab37149; Abcam) for nuclear protein, PERK (1:1 000, 2469 R; Bioss), p-PERK (1:1 000, 3330 R; Bioss), IRE1α (1:1 000, 3294; Cell Signaling Technology), p-IRE1α (1:1 000, 16698 R; Bioss), GAPDH (1:3 000, 60004-1-Ig; ProteinTech), Lamin B1 (1:1 000, AF5161; Affinity Biosciences) and β-actin (1:2 000, 4970; Cell Signaling Technology). Then, the membranes were incubated with HRP-conjugated secondary antibodies. Enhanced chemiluminescence (ECL) reagents (Tanon) were used for chemo-luminescence development, visualized by Tanon-5200 Multi Chemiluminescent System (Tanon). The quantitation of western blot was performed by using ImageJ software. Quantities of target proteins were normalized to that of GAPDH of the same sample.

### RNA interference and Plasmid DNA transfection

For each siRNA experiment, three independent sequences were used to knock down the target genes and the most effective one or two sequences were chosen for further experiments. The selected siRNA sequences of mouse ATF6 were as follows: Forward, 5′-CCUUGGGAGUCAGACCUAUTT-3′, Reverse, 5′-AUAGGUCUGACUCCCAAGGTT-3′; Forward, 5′-GCCACUUCUGCUCAGACAUTT-3′, Reverse, 5′-AUGUCUGAGCAGAAGUGGCTT-3′. A scrambled siRNA was used as a negative control. BMDMs were seeded in Opti-MEM (31985088; Gibco) without serum one day before transfection, then transiently transfected with siRNA using Lipofectamine 2000 Transfection Reagent (11668019; Invitrogen). An overexpression plasmid for mouse ATF6 listed in Supplemental Table III was constructed for further experiments and transfected using Lipofectamine 2000 Transfection Reagent according to the manufacturer’s protocol. 100 nmol/L siRNA or 2 μg/mL plasmid mixture were added for 24-48 h before collection for experiments.

### RNA sequencing

Total RNA from each sample was extracted using TRIzol Reagent (15596026; Invitrogen). The concentration and integrity of RNA were determined using an Agilent 2100 Bioanalyzer (Agilent Technologies) with RIN number >7.0. Then the cleaved RNA fragments were reverse-transcribed to create the cDNA by SuperScript™ II Reverse Transcriptase (1896649; Invitrogen). Dual-index adapters were ligated to the fragments, and size selection was performed with AMPureXP beads. The ligated products were enriched with 8 cycles of PCR to create the final cDNA library. The RNA libraries were sequenced on the illumina Novaseq™ 6000 (LC Bio Technology CO., Ltd.,Hangzhou, China). The RNA-seq data have been deposited in the Gene Expression Omnibus (GEO) datasets under accession code GSE246048.

The original sequence data was aligned and annotated referring to the mouse reference genome GRCm39. The FPKM value was used to estimate the gene expression level, and edgeR and Q value were used to identify the differential gene expression between the two groups. Thereafter, the differences of gene abundance between samples were calculated based on the ratio of the FPKM values. The false discovery rate (FDR) control method was used to determine the threshold of the Q value in multiple tests in order to calculate the significance of the differences. The genes with an absolute value of log2 ratio ≥ 1 and Q value < 0.05 were used for subsequent analysis.

### CUT&Tag analysis

CUT&Tag were prepared and analyzed as previously described.^60^ The Hyperactive pA/G-Tn5 Transposase (TD903, Vazyme) and the following primary antibodies were used: anti-ATF6 antibody (ab227830, Abcam). The library preparations were sequenced on Illumina Novaseq platform and 150 bp paired-end reads were generated. Clean reads were aligned to the reference genome using BWA mem v0.7.12 (Burrows Wheeler Aligner). A peak was determined as different peak when the odds ratio between two groups was more than 2. Using the same method, genes associated with different peaks were identified and also do KEGG enrichment analysis. Data analysis and visualization were performed using R scripts. These data have been deposited in the Gene Expression Omnibus (GEO) datasets under accession code GSE245975.

### Protein-DNA docking assay

3D structure model of ATF6 p50 protein was predicted through SWISS-MODEL. DNA 3D modeling and protein-DNA docking steps were consistent with previous reports.^61,62^ PyMOL v2.5.4 was employed for polar interactions analysis and 3D interactions mapping after docking.

### Chromatin immunoprecipitation (ChIP) assay

ChIP assay was performed using a commercial chromatin IP Kit (17-10085, Millipore), following the instructions provided by the manufacturer. Briefly, the ChIP assay was performed using magnetic protein A/G beads and an anti-ATF6 antibody (ab227830, Abcam) or normal rabbit IgG antibody as negative control. The immunoprecipitated DNA was used to amplify DNA fragments via PCR with specific primers. The primer sequences for *Tnfα* promoter were 5′- CCTGCTCAGTAAGGGAGACC-3′ (forward) and 5′-GTCCGACCTAGACCCACAAA -3′ (reverse).

### Construction of reporter plasmids and dual-luciferase reporter assay

Luciferase reporter plasmids containing wild-type and mutant *Tnfα* promoters were inserted into the pGL4.31 vector (Supplemental Table IV). The corresponding ATF6 mutants were cloned and inserted into the pcDNA3.1 plasmid (Supplemental Table V). 293 T cells were co-transfected with mAtf6-pcDNA3.1 and mTnfα-pGL4.31 vectors using Lipofectamine 3000 (Invitrogen). After 48 h, luciferase activity was measured using the Dual-Luciferase Reporter Assay System (Promega) and normalized to that of renilla to determine the promoter activity. Results were expressed as fold changes of control group.

### Statistical analysis

All quantitative data were expressed as the mean ± SD based on at least three independent samples. GraphPad Prism 8.0 (Graph Pad Prism Software, Inc, San Diego, CA) was used for statistical analysis. The Student’s t-test was used for statistical comparisons of 2 groups and statistical comparisons of multiple groups were performed by one-way ANOVA. *P* < 0.05 was considered to indicate a statistically significant difference.

## Supplementary information

SUPPLEMENTAL MATERIAL

## Data Availability

The data used to support the findings of this study are available from the corresponding author upon request.
