# Supplementary material for: Macrophage ATF6 accelerates corticotomy-assisted orthodontic tooth movement through promoting Tnfα transcription
Source: Int J Oral Sci. 2025 Apr 1;17:28. doi: 10.1038/s41368-025-00359-7 (PMC11958779; doi:10.1038/s41368-025-00359-7)
Supplement: Supplementary file 1 — SUPPLEMENTAL MATERIAL [file 41368_2025_359_MOESM1_ESM.pdf]

# Supplemental Figure 1

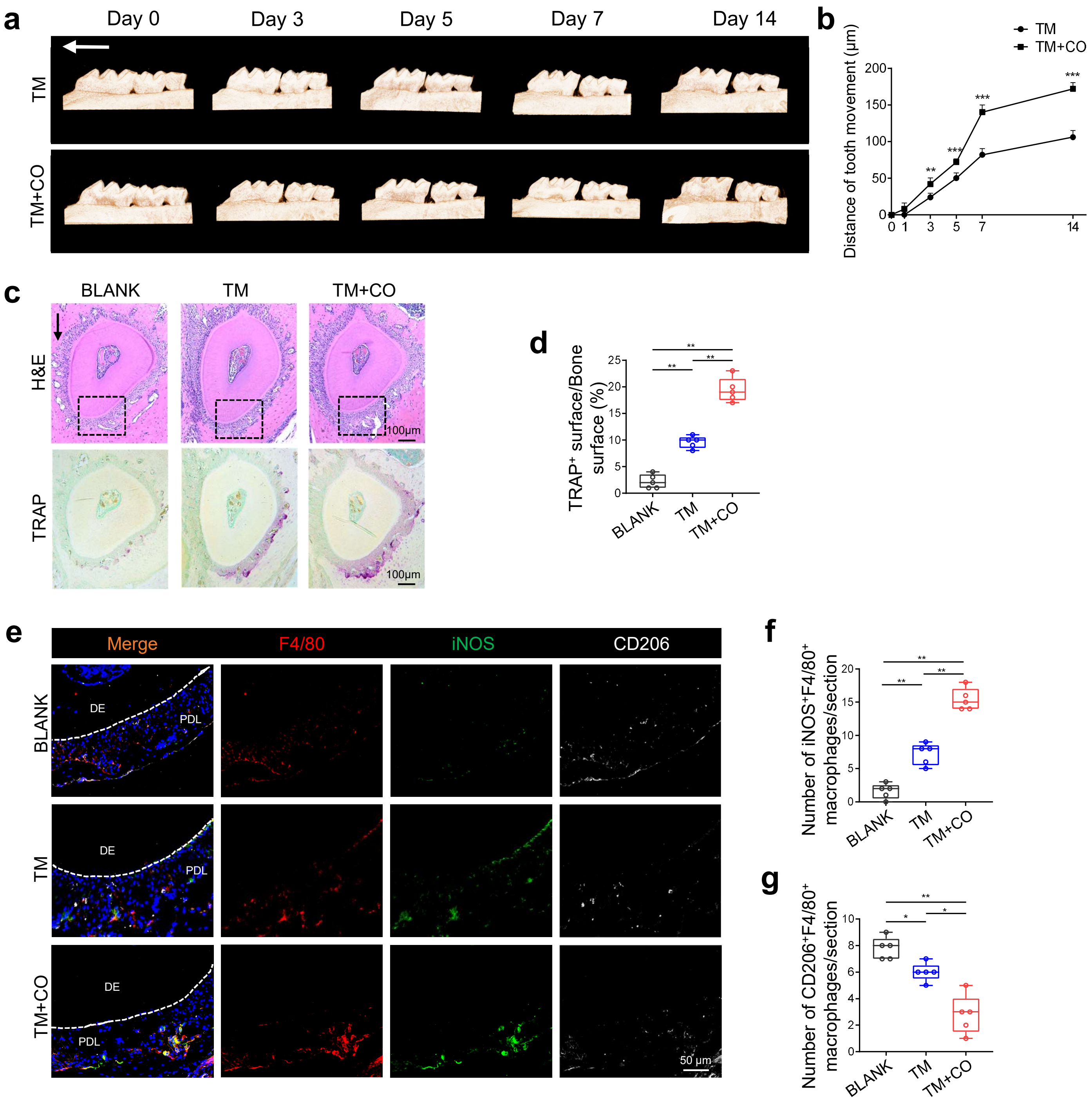


**Supplemental Figure 1. Corticotomy induces pro-inflammatory macrophage polarization.**
(**a, b**) Representative 3D reconstruction images and statistics of the tooth movement distance from Day 0 to Day 14 in TM and TM+CO groups.
(**c**) Representative H&E staining and TRAP staining of murine first molar after 7-day application of orthodontic force and corticotomy. Scale bars, 100 μm.
(**d**) Quantification of surface of osteoclasts relative to the bone surface was shown.
(**e-g**) Representative immunofluorescence staining images (**e**) and quantification of iNOS$^{+}$ (green) F4/80$^{+}$ (red) cells (**f**) and CD206$^{+}$ (white) F4/80$^{+}$ (red) cells (**g**) in murine periodontal tissues from BLANK, TM and TM+CO groups. Scale bars, 50 μm.
Data represent mean ± SD. N = 5. * $P < .05$, ** $P < .01$. TM, tooth movement; TM+CO, corticotomy-assisted orthodontic tooth movement.

# Supplemental Figure 2

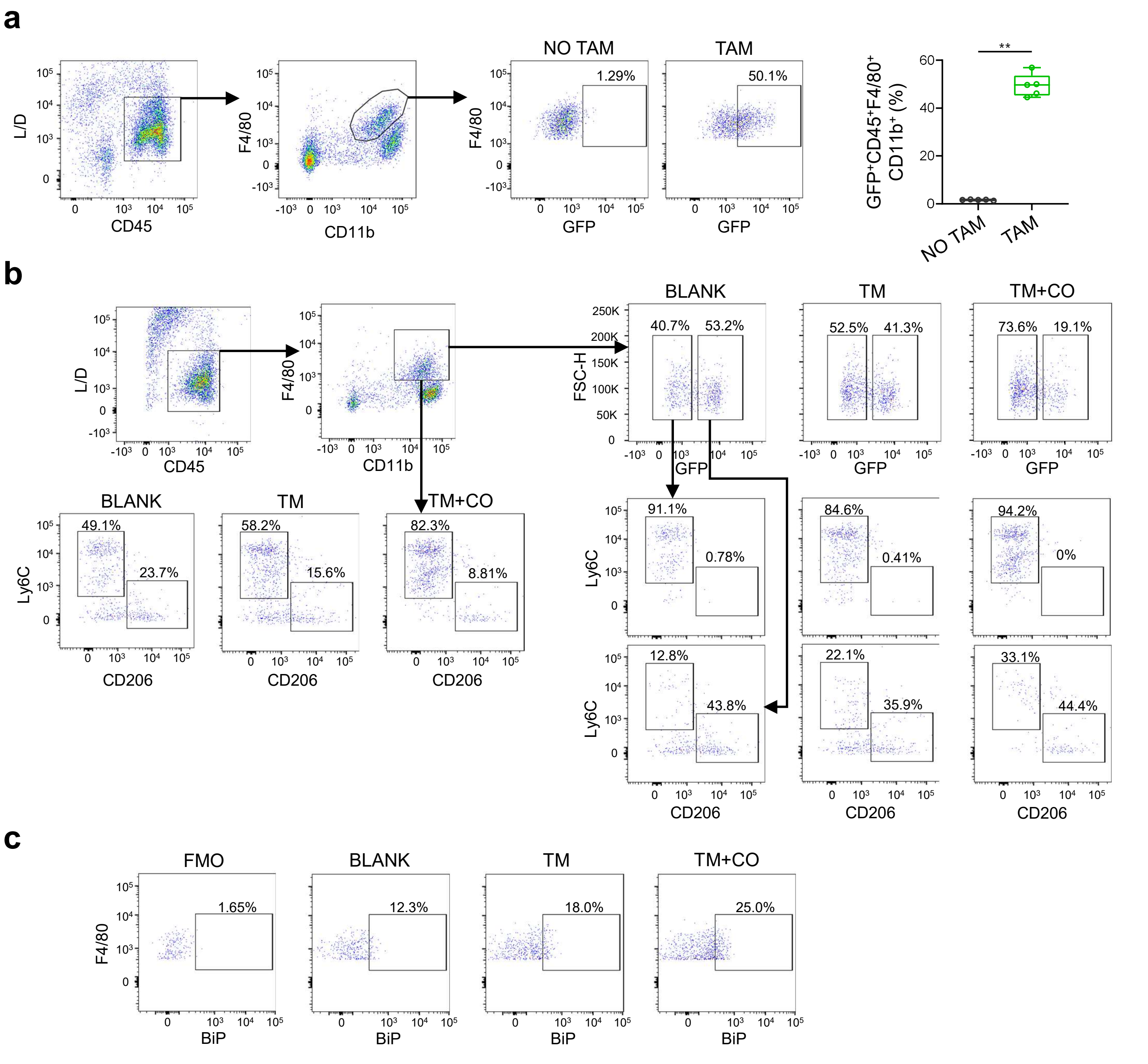


**Supplemental Figure 2. Gating strategies of FACS analysis.**

(**a**) Gating strategies of $GFP^+CD45^+F4/80^+CD11b^+$ macrophages in alveolar bone tissues from $CX3CR1^{CreERT2}$; $R26^{GFP}$ mice with or without tamoxifen injection.

(**b**) Gating strategies of $CD45^+F4/80^+CD11b^+Ly6C^+$ pro-inflammatory and $CD45^+F4/80^+CD11b^+CD206^+$ reparative macrophages, $GFP^-CD45^+F4/80^+CD11b^+$ monocyte-derived and $GFP^+CD45^+F4/80^+CD11b^+$ resident macrophages from BLANK, TM and TM+CO groups.

(**c**) Gating strategies of $BiP^+GFP^-CD45^+F4/80^+CD11b^+$ monocyte-derived macrophages from BLANK, TM and TM+CO groups.

Data represent mean ± SD. N = 5. ** $P < .01$.

# Supplemental Figure 3

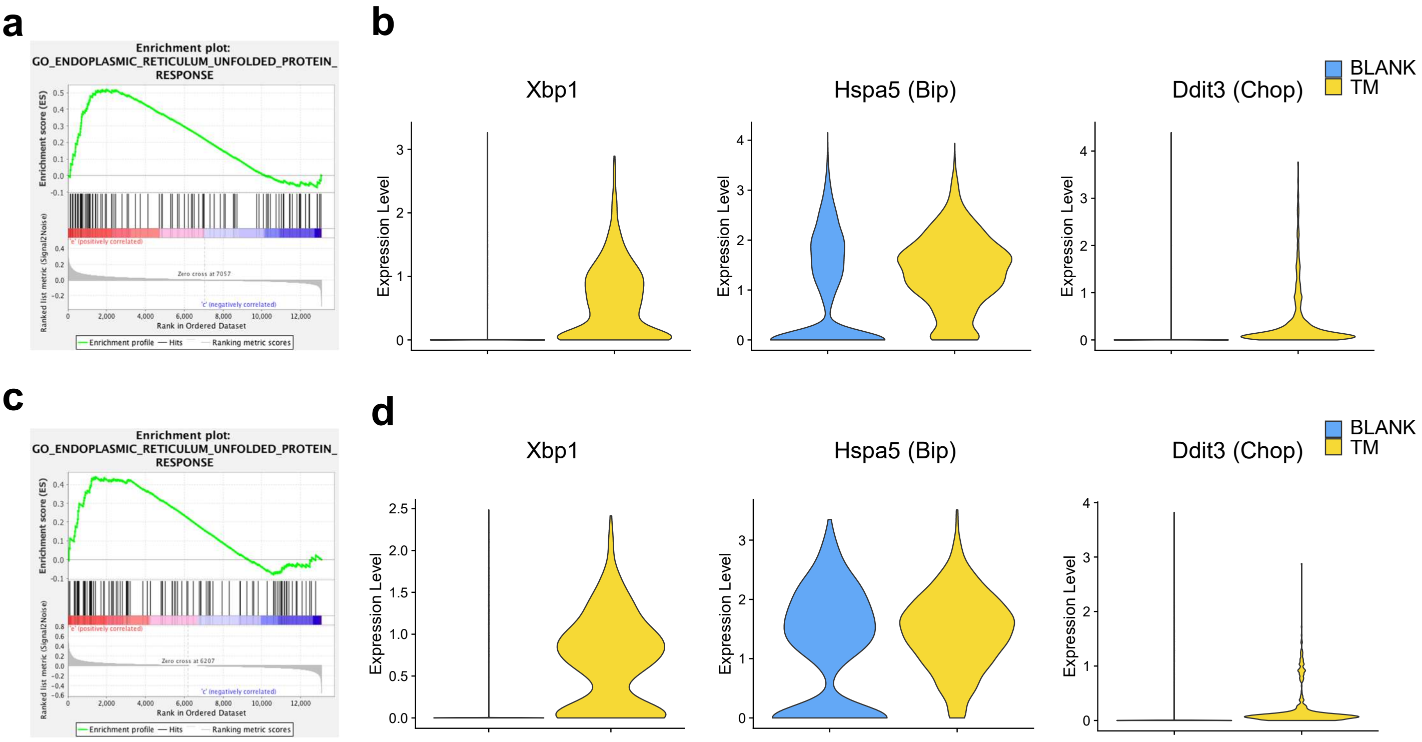


**Supplemental Figure 3. UPR is activated in macrophages during orthodontic tooth movement.**
(**a**) Gene set enrichment analysis (GSEA) of the distribution of genes in Endoplasmic Reticulum Unfolded Protein Response gene sets in total macrophages from BLANK and TM groups.
(**b**) Violin plots of the expression level of UPR marker genes *Xbp1*, *Hspa5* and *Ddit3* in total macrophages from BLANK and TM groups.
(**c**) Gene set enrichment analysis (GSEA) of the distribution of genes in Endoplasmic Reticulum Unfolded Protein Response gene sets in $CCR2^{+}$ monocyte-derived macrophages from BLANK and TM groups.
(**d**) Violin plots of the expression level of UPR marker genes *Xbp1*, *Hspa5* and *Ddit3* in $CCR2^{+}$ monocyte-derived macrophages from BLANK and TM groups.

# Supplemental Figure 4

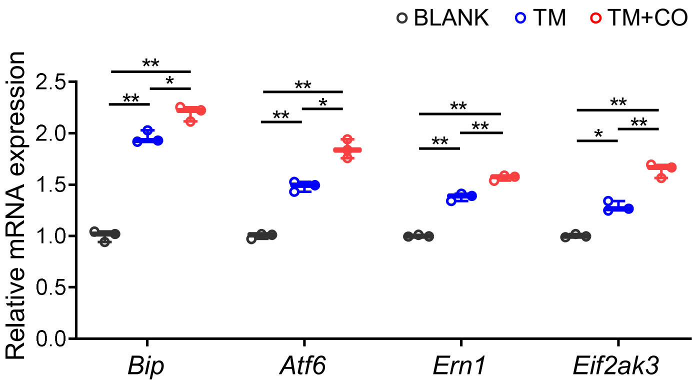


**Supplemental Figure 4. RT-PCR analysis of *Bip*, *Atf6*, *Ern1* and *Eif2ak3* mRNA expression in BMDMs with stimulation of alveolar bone lysates from Blank, TM and TM+CO mice.**
Data represent mean ± SD. N = 3. * P < .05, ** P < .01. TM, tooth movement; TM+CO, corticotomy-accelerated orthodontic tooth movement.

# Supplemental Figure 5

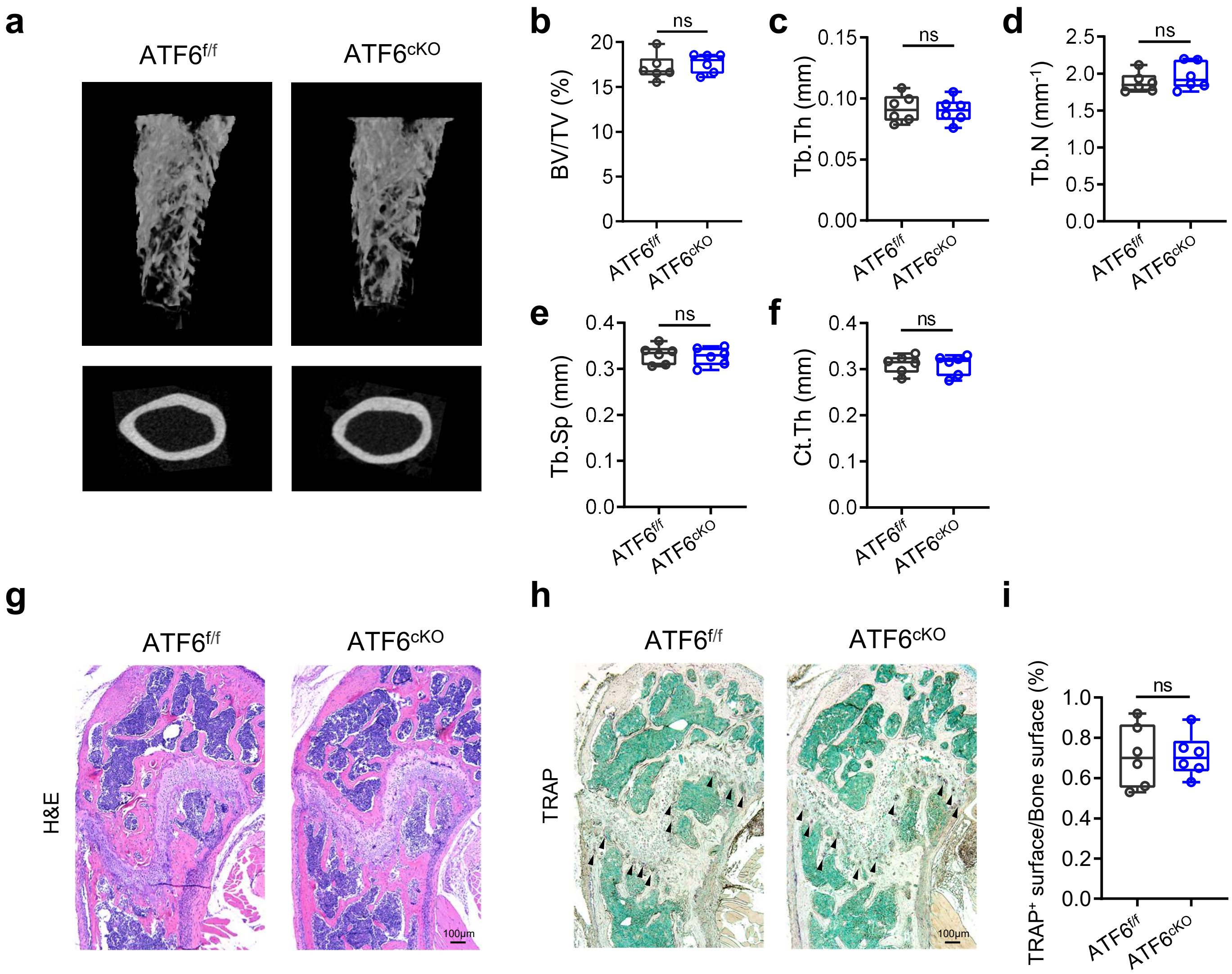


**Supplemental Figure 5. Macrophage-specific ATF6 deletion has no significant effect on general bone phenotypes in mice.**

(**a**) Representative 3D reconstruction images of distal femurs in $ATF6^{f/f}$ or $ATF6^{cKO}$ mice.

(**b-f**) Quantitative parameters of micro-CT, including bone volume relative to tissue volume (**b**), trabecular thickness (**c**), trabecular number (**d**), trabecular separation (**e**) and cortical thickness (**f**) were shown.

(**g, h**) Representative H&E (**g**) and TRAP staining (**h**) images of distal femurs in $ATF6^{f/f}$ or $ATF6^{cKO}$ mice. Scale bars of H&E staining images and TRAP staining images, 100 μm.

(**i**) Quantification of surface of osteoclasts relative to the bone surface was shown.

Data represent mean ± SD. N = 6. ns, no significance.

# Supplemental Figure 6

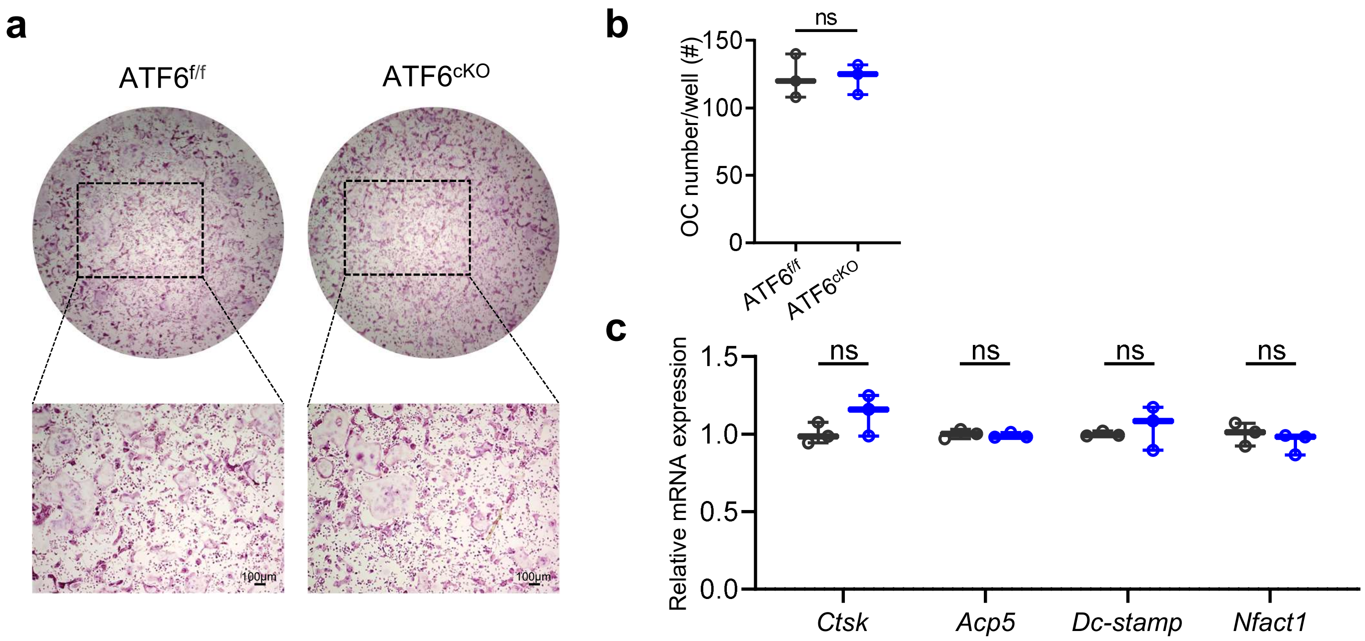


**Supplemental Figure 6. Macrophage ATF6 deletion does not affect osteoclast differentiation and function in vitro.**

(**a, b**) Representative TRAP staining images (**a**) and quantification (**b**) of multinuclear osteoclasts from ATF6$^{f/f}$ or ATF6$^{cKO}$ mice at 5 days cultured in M-CSF (25 ng/mL) and RANKL (10 ng/mL). Scale bar, 100 μm.

(**c**) RT-PCR analysis of *Ctsk*, *Acp5*, *Dc-stamp* and *Nfatc1* mRNA expression in osteoclasts from ATF6$^{f/f}$ or ATF6$^{cKO}$ mice at 5 days cultured in M-CSF (25 ng/mL) and RANKL (10 ng/mL).

Data represent mean ± SD. N = 3. ns, no significance.

# Supplemental Figure 7

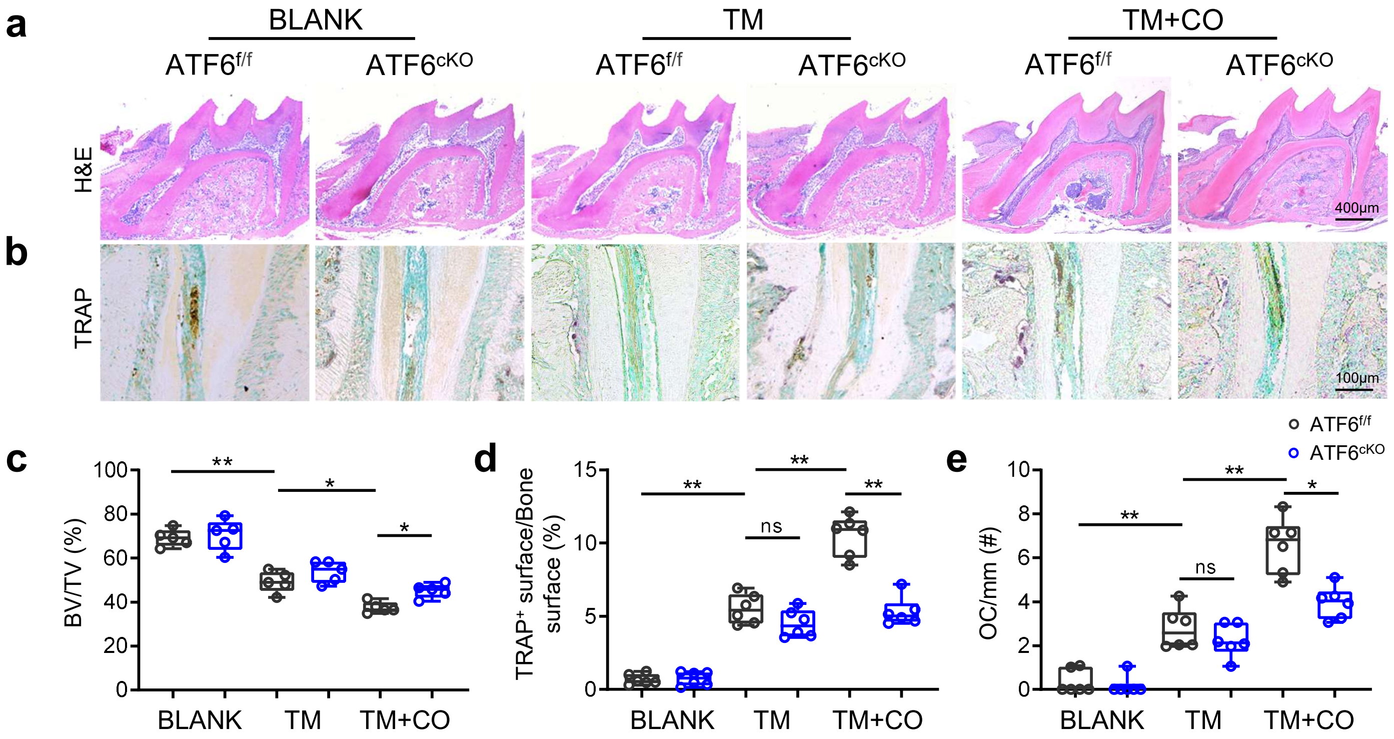


**Supplemental Figure 7. Macrophage ATF6 deficiency suppresses orthodontic bone remodeling in corticotomy.**

(**a, b**) Representative H&E (**a**) and TRAP staining (**b**) images of murine first molar after 7-day application of orthodontic force and corticotomy in ATF6$^{f/f}$ or ATF6$^{cKO}$ mice. Scale bars of H&E staining images, 400 μm. Scale bars of TRAP staining images, 100 μm.

(**c-e**) Quantification of bone volume relative to tissue volume (**c**), surface of osteoclasts relative to the bone surface (**d**) and number of TRAP-positive osteoclasts relative to bone surface (**e**) were shown.

Data represent mean ± SD. N = 6. * $P < .05$, ** $P < .01$. ns, no significance. TM, tooth movement; TM+CO, corticotomy-accelerated orthodontic tooth movement.

# Supplemental Figure 8

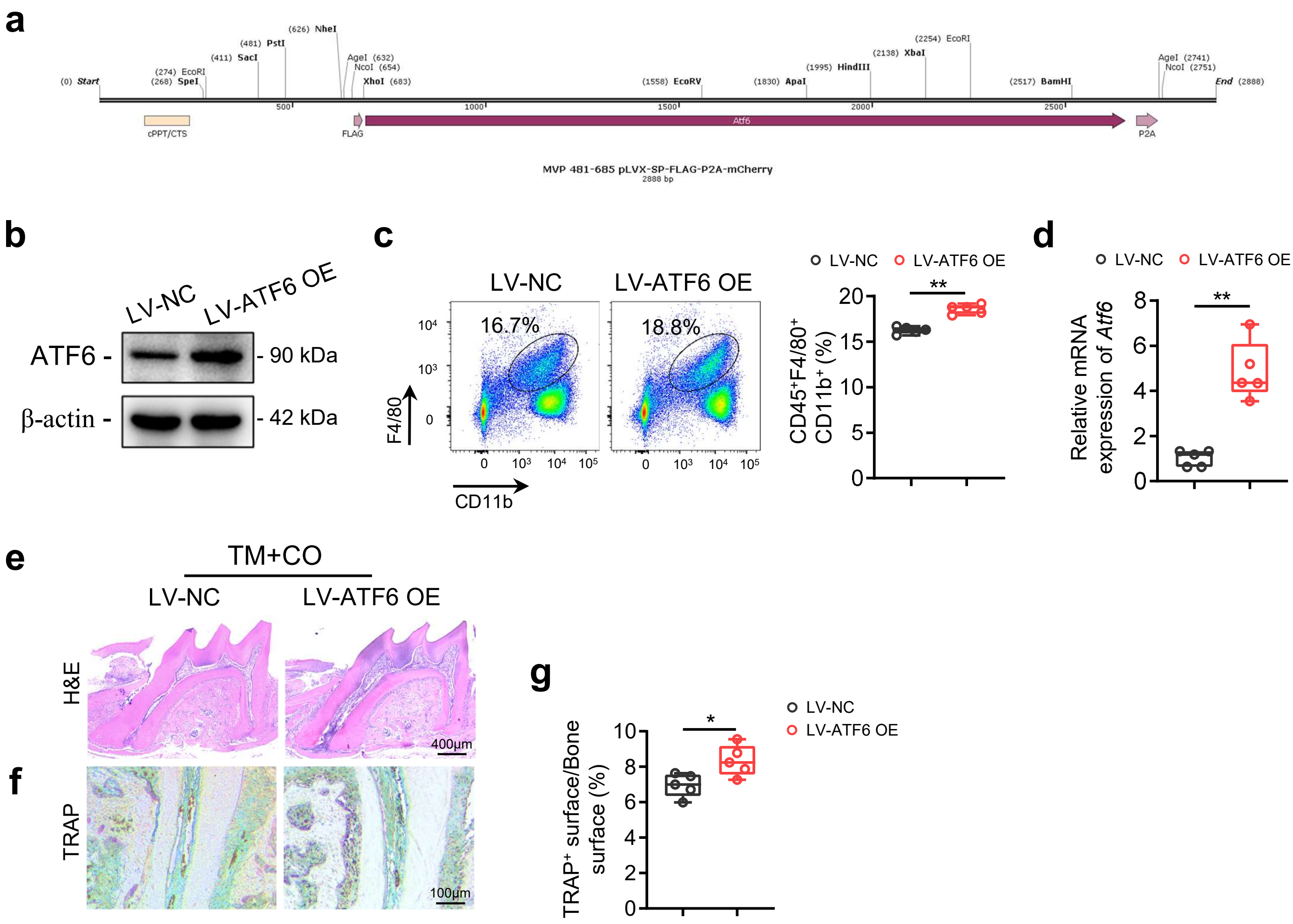


**Supplemental Figure 8. Confirmation of macrophage-specific Atf6-gene overexpressed lentivirus.**

(**a**) Schematic diagram of vector of macrophage-specific Atf6-gene overexpressed lentivirus.

(**b**) Representative western blotting of ATF6 expression in BMDMs treated with Atf6-gene overexpressed lentivirus or negative control.

(**c**) Representative flow plots of the gating scheme used to sort alveolar bone macrophages from mice injected with overexpression lentivirus or negative control.

(**d**) RT-PCR analysis of ATF6 mRNA expression in sorted macrophages.

(**e, f**) Representative H&E staining(**e**) and TRAP staining (**f**) images of murine first molar in TM+CO mice with overexpression lentivirus injection. Scale bars of H&E staining images, 400 µm. Scale bars of TRAP staining images, 100 µm.

(**g**) Quantification of surface of osteoclasts relative to the bone surface was shown.

Data represent mean ± SD. N = 5. * $P < .05$, ** $P < .01$. ns, no significance. TM+CO, corticotomy-accelerated orthodontic tooth movement; LV-ATF6 OE, ATF6-gene-overexpressed lentivirus; LV-NC, scrambled lentivirus served as a negative control.

# Supplemental Figure 9

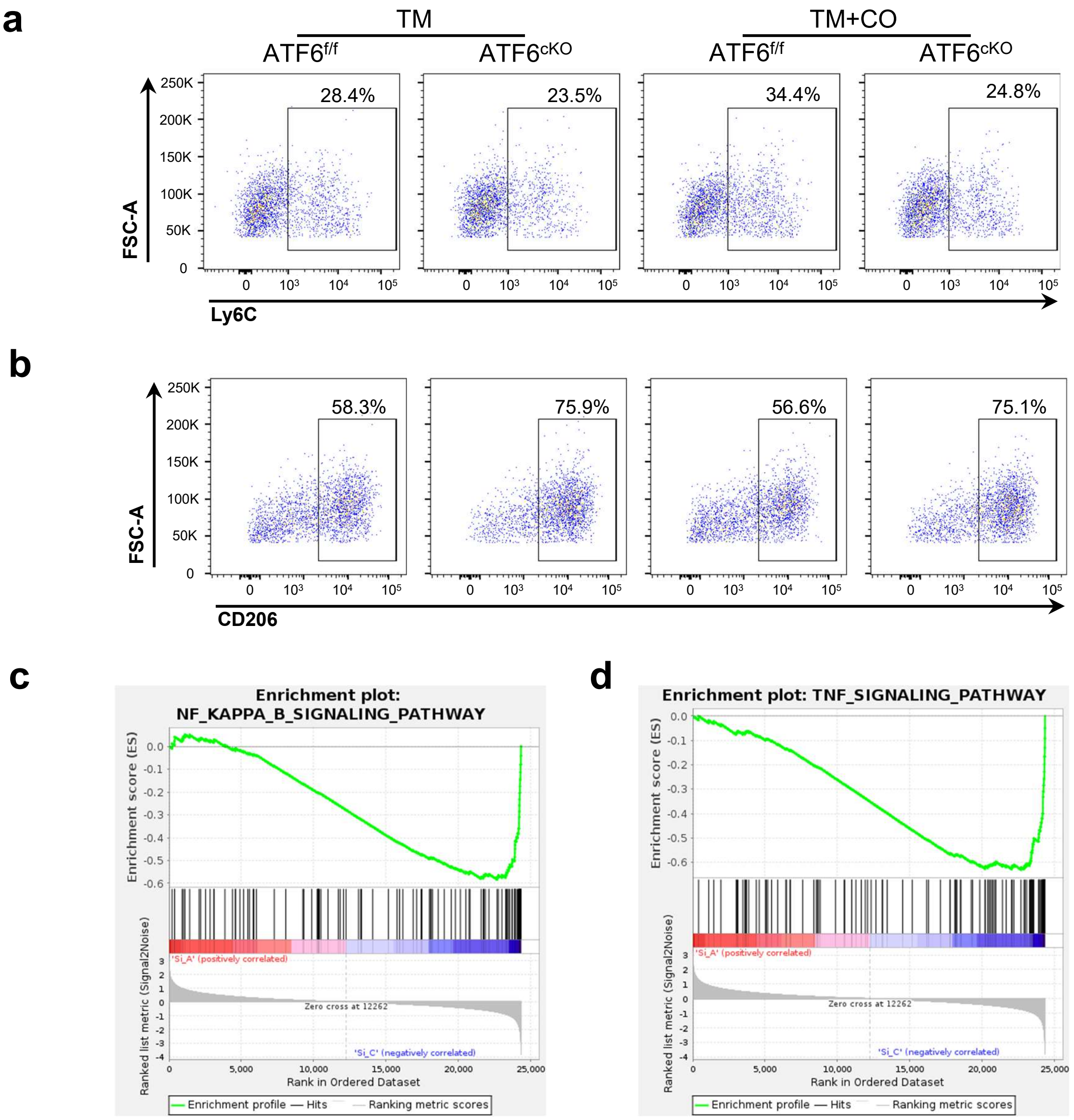


**Supplemental Figure 9. ATF6 promotes macrophage pro-inflammatory signals in vitro.**

(**a**) Gating strategies of $CD45^{+}F4/80^{+}CD11b^{+}Ly6C^{+}$ macrophages in BMDMs from $ATF6^{f/f}$ or $ATF6^{cKO}$ mice upon stimulation of alveolar bone lysates from TM or TM+CO mice.

(**b**) Gating strategies of $CD45^{+}F4/80^{+}CD11b^{+}CD206^{+}$ macrophages in BMDMs from $ATF6^{f/f}$ or $ATF6^{cKO}$ mice upon stimulation of alveolar bone lysates from TM or TM+CO mice.

(**c, d**) Gene set enrichment analysis (GSEA) of the distribution of genes in the NF-κB signaling pathway (**c**) and TNF signaling pathway (**d**) gene sets of ATF6 knockdown and control groups.

# Supplemental Figure 10

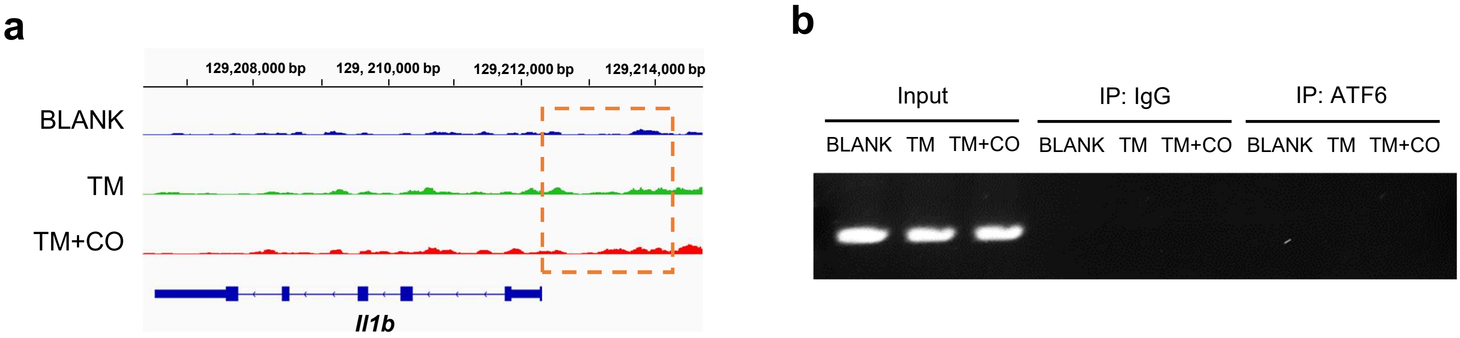


**Supplemental Figure 10. ATF6 p50 did not mediate the transcription of *Il1b*.**
(**a**) IGV snapshot of peak values of ATF6 p50 binding on *Il1b* gene in BMDMs from Blank, TM and TM+CO groups.
(**b**) ChIP assay of the binding of ATF6 p50 to *Il1b* promoter in BMDMs treated with tissue suspension from Blank, TM and TM+CO group.

# Supplemental Figure 11

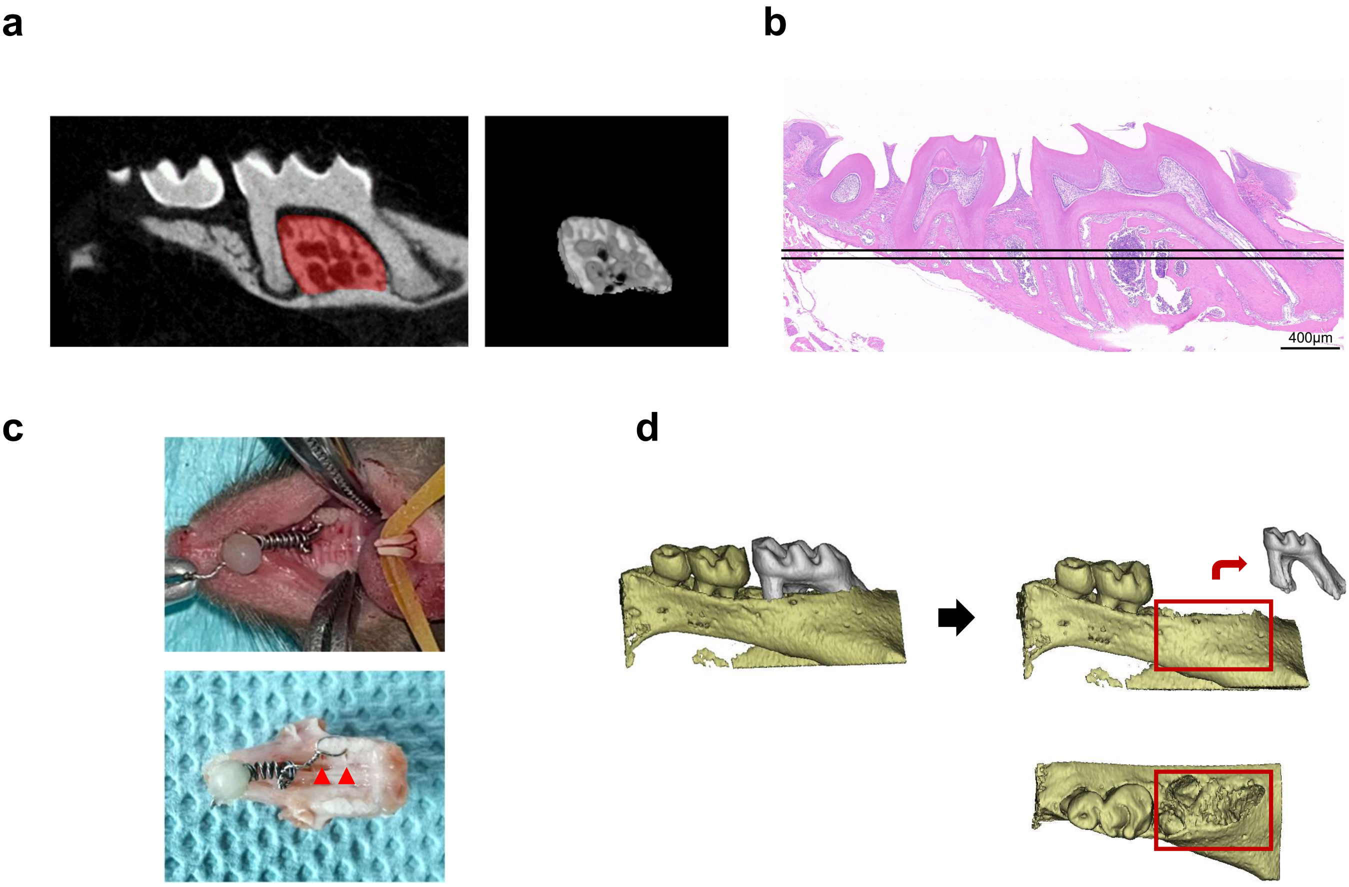


**Supplemental Figure 11. Schematic representation of animal models construction, tissue lysates sampling sites, tissue section preparation sites and the region of interest for volumetric measurements.**

(**a**) Representative 3D reconstruction images of the region of interest for volumetric measurements.

(**b**) Sections were obtained from the middle part (between the double black bars). Scale bars of H&E staining images, 400 μm.

(**c**) Representative 3D reconstruction images and pictures of the TM+CO models.

(**d**) Schematic illustration of tissue lysates sampling sites.

**Supplemental Table I.** **The mouse Atf6 pLV-SP sequence**

mAtf6 pLV-SP sequence:
CCCACCTTCCCAACTCTCTAGGGGACCCGACAGGCCCGAAGGAATAGAAGAAGAAGGTGGAGAGAGAGACAGAGACAGATCC
ATTCGATTAGTGAACGGATCTCGACGGTATCGCCTTTAAAAGAAAAGGGGGGATTGGGGGGTACAGTGCAGGGGAAAGAATAGT
AGACATAATAGCAACAGACATACAAACTAAAGAATTACAAAAACAAATTACAAAAATTCAAAATTTTCGGGTTTATTACAGGGACAG
CAGAGATCCAGTTTACTAGTGAATTCAAGCGACTTCCTCTTTCCAGCAGAAAAGGAGAAGTAGGAGCCAAGATTTCCAAACTCTG
TGGTTGCCTTGAAGCGACTTCCTCTTTCCAGAAGCGACTTCCTCTTTCCAGCAGAAAAGGAGAAGTAGGAGCTCCTACTTCTCC
TTTTCTGCCAAGATTTCCAAACTCTGTGGTTGCCTTGCTCCTACTTCTCCTTTTCTGCAGAAAAGGAGAAGTAGGAGCTGGAAAG
AGGAAGTCGCTTTACCCTCCCTGCCCCCTGTCCCGACCGCGACAAAAGCGACTTCCTCTTTCCAGTGCATTTAAGGCGCAGCC
TGGAAGTGCCAGGGAGCACTGGAGGCCACCCAGTCGCTAGCACCGGTCGTTGGCGCGCCGCCACCATGGACTACAAGGACG
ACGATGACAAGCTCGAGATGGAGTCGCCTTTTAGTCCGGTTCTTCCTCATGGACCAGATGAAGACTGGGAGTCTACGTTGTTTG
CTGAACTTGGCTATTTCACAGACACTGATGATGTGCACTTTGATGCAGCACATGAGGCTTATGAAAATAATTTTGATCATCTTAATT
TTGATTTGGATTTGATGCCTTGGGAGTCAGACCTATGGAGCCCCGGCAGCCACTTCTGCTCAGACATGAAGGCAGAGCCCCAG
CCTCTTTCTCCGGCTTCCTCCAGTTGCTCCATCTCCTCTCCTCGGTCCACAGACTCGTGTTCTTCAACTCAGCACGTTCCTGAG
GAGTTGGATTTGTTGTCTAGTTCTCAGTCCCCCCTTTCCTTATATGGCGACAGCTGTAATAGCCCCTCCTCTGTAGAGCCACTGA
AGGAAGAGAAGCCTGTCACTGGTCCTGGAAACAAAACAGAACATGGACTGACTCCAAAGAAAAAAATTCAGATGAGTTCAAAAC
CTTCAGTTCAGCCCAAGCCTTTATTACTTCCAGCAGCGCCCAAGACTCAAACCAATGCCAGTGTCCCAGCAAAAGCCATCATCAT
TCAGACACTACCAGCCCTTATGCCACTGGCAAAGCAGCAGTCGATTATCAGCATACAGCCTGCGCCCACCAAAGGCCAGACTGT
TTTGCTCTCTCAGCCGACTGTGGTTCAACTTCAGAGCCCTGCGGTTCTGTCGTCTGCTCAGCCGGTTCTCGCAGTCACTGGGG
GAGCCGCACAGCTGCCTAACCATGTGGTGAATGTGCTGCCAGCCCCTGTGGTGAGCAGCCCGGTGAATGGAAAACTCTCCGT
GACTAAACCTGTTCTACAAAGTGCCACCAGAAGTATGGGTTCGGATATCGCTGTGCTGAGGAGACAGCAGCGGATGATAAAGAA
CCGAGAGTCTGCTTGTCAGTCGCGCAAGAAGAAGAAAGAGTATATGCTAGGACTGGAGGCCAGGCTCAAGGCTGCCCTCTCAG
AGAATGAGCAGCTGAAGAAGGAGAATGGCTCCCTGAAGCGACAGCTGGACGAGGTGGTGTCAGAGAACCAGAGGCTCAAAGT
CCCAAGTCCAAAGCGAAGAGCTGTCTGTGTGATGATAGTATTAGCATTTATAATGCTGAACTATGGGCCCATGAGCATGCTGGAG
CAAGAGTCCCGAAGAGTGAAACCTAGTGTGAGCCCTGCCAATCAGAGGAGGCATCTCTTGGAATTTTCAGCGAAAGAAGTTAAA
GACACATCCGATGGTGACAACCAGAAAGACAGTTACAGCTATGATCACTCTGTGTCCAATGACAAAGCTTTAATGGTGCTAAGTG
AAGAGCCATTGCTTTATATGCCTCCACCTCCATGTCAACCCCTGATTAACACGACAGAGTCTCTCAGGTTGAACCATGAACTTCG
AGGCTGGGTTCATAGACATGAAGTGGAAAGGACCAAATCTAGAAGAATGACAAATAGCCAACAGAAAGCCCGCATTCTCCAGGG
TGCTCTGGAACAGGGCTCTAATTCTCAGCTGATGGCTGTCCAGTACACAGAAACCACTAGCATCAGTAGGAATTCTGGGAGTGA
GCTGCAAGTGTATTACGCCTCCCCTGGAAGTTACCAAGGCTTCTTTGACGCCATCCGCAGGAGGGGAGATACGTTTTACGTGGT
CTCATTTCGAAGGGATCATCTGCTATTACCAGCTACCACCCACAACAAGACCACAAGACCAAAAATGTCAATTGTATTACCAGCAA
TAAACATAAATGATAATGTGATCAATGGGCAGGACTATGAAGTGATGATGCAGATTGACTGTCAGGTGATGGACACCAGGATCCT
CCACATCAAAAGCTCCTCAGTCCCCCCTTATCTCCGGGATCATCAGCGGAACCAAACCAGCACCTTCTTTGGTTCCCCTCCAAC
AACCACAGAGACGACCCATGTGGTCAGCACCATCCCTGAGTCGTTGCAGCGGATCAGAGCCAAGAGAGGAAGCGGAGCTACT
AACTTCAGCCTGCTGAAGCAGGCTGGAGACGTGGAGGAGAACCCTGGACCTACCGGTGCCACCATGGTGAGCAAGGGCGAG
GAGGATAACATGGCCATCATCAAGGAGTTCATGCGCTTCAAGGTGCACATGGAGGGCTCCGTGAACGGCCACGAGTTCGAGAT
CGAGGGCGAGGGCGAGCCGCCCTACGAGCCCCGCG

**Supplemental Table II.** **RT-PCR primers used in this study.**

| Gene Name | Forward primer 5'-3' | Reverse primer 5'-3' |
|---|---|---|
| Eif2ak3 | GGGAAAACGGTTCTGAGACA | GCTGACCAGCTAGTCTTGGG |
| Ern1 | AACACACCGACCACCGTATC | AGGGTCCTGGGTAAGGTCTC |
| Atf6 | GAACTTCGAGGCTGGGTTCA | TCCAGGGGAGGCGTAATACA |
| Bip | CAGATCTTCTCCACGGCT | TGTCACTCGGAGAATACCAT |
| Tnfα | GTTCTATGGCCCAGACCCTCAC | GGCACCACTAGTTGGTTGTCTTTG |
| Il1β | TCCAGGATGAGGACATGAGCAC | GAACGTCACACACCAGCAGGTTA |
| Il6 | CCACTTCACAAGTCGGAGGCTTA | GCAAGTGCATCATCGTTGTTCATAC |
| Ccl2 | AGCAGCAGGTGTCCCAAAGA | GTGCTGAAGACCTTAGGGCAGA |
| Cd206 | CTCTGTTCAGCTATTGGACGC | CGGAATTTCTGGGATTCAGCTTC |
| Arg1 | GCTCAGGTGAATCGGCCTTTT | TGGCTTGCGAGACGTAGAC |
| Il10 | GCCCTTTGCTATGGTGTCCTTTC | TCCCTGGTTTCTCTTCCCAAGAC |
| Gapdh | AGGTCGGTGTGAACGGATTTG | TGTAGACCATGTAGTTGAGGTCA |

# Supplemental Table III. The mouse Atf6 sequence

mouse Atf6 sequence：1971bp

ATGGAGTCGCCTTTTAGTCCGGTTCTTCCTCATGGACCAGATGAAGACTGGGAGTCTACGTTGTTTGCTGAACTTGGCTATTTCA
CAGACACTGATGATGTGCACTTTGATGCAGCACATGAGGCTTATGAAAATAATTTTGATCATCTTAATTTTGATTTGGATTTGATGC
CTTGGGAGTCAGACCTATGGAGCCCCGGCAGCCACTTCTGCTCAGACATGAAGGCAGAGCCCCAGCCTCTTTCTCCGGCTTCC
TCCAGTTGCTCCATCTCCTCTCCTCGGTCCACAGACTCGTGTTCTTCAACTCAGCACGTTCCTGAGGAGTTGGATTTGTTGTCTA
GTTCTCAGTCCCCCCTTTCCTTATATGGCGACAGCTGTAATAGCCCCTCCTCTGTAGAGCCACTGAAGGAAGAGAAGCCTGTCA
CTGGTCCTGGAAACAAAACAGAACATGGACTGACTCCAAAGAAAAAAATTCAGATGAGTTCAAAACCTTCAGTTCAGCCCAAGC
CTTTATTACTTCCAGCAGCGCCCAAGACTCAAACCAATGCCAGTGTCCCAGCAAAAGCCATCATCATTCAGACACTACCAGCCCT
TATGCCACTGGCAAAGCAGCAGTCGATTATCAGCATACAGCCTGCGCCCACCAAAGGCCAGACTGTTTTGCTCTCTCAGCCGAC
TGTGGTTCAACTTCAGAGCCCTGCGGTTCTGTCGTCTGCTCAGCCGGTTCTCGCAGTCACTGGGGGAGCCGCACAGCTGCCTA
ACCATGTGGTGAATGTGCTGCCAGCCCCTGTGGTGAGCAGCCCGGTGAATGGAAAACTCTCCGTGACTAAACCTGTTCTACAAA
GTGCCACCAGAAGTATGGGTTCGGATATCGCTGTGCTGAGGAGACAGCAGCGGATGATAAAGAACCGAGAGTCTGCTTGTCAG
TCGCGCAAGAAGAAGAAAGAGTATATGCTAGGACTGGAGGCCAGGCTCAAGGCTGCCCTCTCAGAGAATGAGCAGCTGAAGAA
GGAGAATGGCTCCCTGAAGCGACAGCTGGACGAGGTGGTGTCAGAGAACCAGAGGCTCAAAGTCCCAAGTCCAAAGCGAAGA
GCTGTCTGTGTGATGATAGTATTAGCATTTATAATGCTGAACTATGGGCCCATGAGCATGCTGGAGCAAGAGTCCCGAAGAGTGA
AACCTAGTGTGAGCCCTGCCAATCAGAGGAGGCATCTCTTGGAATTTTCAGCGAAAGAAGTTAAAGACACATCCGATGGTGACA
ACCAGAAAGACAGTTACAGCTATGATCACTCTGTGTCCAATGACAAAGCTTTAATGGTGCTAAGTGAAGAGCCATTGCTTTATATG
CCTCCACCTCCATGTCAACCCCTGATTAACACGACAGAGTCTCTCAGGTTGAACCATGAACTTCGAGGCTGGGTTCATAGACATG
AAGTGGAAAGGACCAAATCTAGAAGAATGACAAATAGCCAACAGAAAGCCCGCATTCTCCAGGGTGCTCTGGAACAGGGCTCTA
ATTCTCAGCTGATGGCTGTCCAGTACACAGAAACCACTAGCATCAGTAGGAATTCTGGGAGTGAGCTGCAAGTGTATTACGCCTC
CCCTGGAAGTTACCAAGGCTTCTTTGACGCCATCCGCAGGAGGGGAGATACGTTTTACGTGGTCTCATTTCGAAGGGATCATCT
GCTATTACCAGCTACCACCCACAACAAGACCACAAGACCAAAAATGTCAATTGTATTACCAGCAATAAACATAAATGATAATGTGAT
CAATGGGCAGGACTATGAAGTGATGATGCAGATTGACTGTCAGGTGATGGACACCAGGATCCTCCACATCAAAAGCTCCTCAGT
CCCCCCTTATCTCCGGGATCATCAGCGGAACCAAACCAGCACCTTCTTTGGTTCCCCTCCAACAACCACAGAGACGACCCATGT
GGTCAGCACCATCCCTGAGTCGTTGCAGTAG

# Supplemental Table IV. The wild-type and mutant Tnfα promoter sequence

mTnfα promoter sequence： 2000bp

TGGAGAAAGCTGCTCCCCCAGGGCCATTCCCACTCCCATCTACCTGGCACACGAGGTCCAGCTCTTTTCCTCCCAATACCCCTTCC
ATGTGCCTCTCCTCAGTGCGCAGAAGTCTGTGTATCCGGGACTTCAAGGACCGTGGGTGCGCTCAATGTACCAGGGGGCTGTGTT
CCTGCTCAGTAAGGGAGACCAGCTGTCCACCCACACCGACGGCATCTCCCATCTACACTTCAGCCCCAGCAGTGTATTCTTTGGAG
CCTTTGCACTGTAGATTCTAAAGAAACCCAAGAATTGGATTCCAGGCCTCCATCCTGACCGTTGTTTCAAGGGTCACATCCCCACAG
TCTCCAGCCTTCCCCACTAAAATAACCTGGAGCTCTCACGGGAGTCTGAGACACTTCAGGGGACTACATCTTCCCCAGGGCCACTC
CAGATGCTCAGGGGACGACTCAAGCCTACCTAGAAGTTCCTGCACAGAGCAGGGTTTTTGTGGGTCTAGGTCGGACAGAGACCTG
GACATGAAGGAGGGACAGACATGGGAGAGGTGGCTGGGAACAGGGGAAGGTTGACTATTTATGGAGAGAAAAGTTAAGTTATTTAT
TTATAGAGAATAGAAAGAGGGGAAAAATAGAAAGCCGTCAGATGACAACTAGGTCCCAGACACAAAGGTGTCTCACCTCAGACAGG
ACCCATCTAAGAGAGAGATGGCGAGAGAATTAGATGTGGGTGACCAAGGGGTTCTAGAAGAAAGCACGAAGCTCTAAAAGCCAGC
CACTGCTTGGCTAGACATCCACAGGGACCCCCTGCACCATCTGTGAAACCCAATAAACCTCTTTTCTCTGAGATTCTGTCTGCTTGT
GTCTGTCTTGCGTTGGGGGAGAAACTTCCTGGTCTCTTTAAGGAGTGGAGCAGGGGACAGAGGCCTCAGTTGGTCCATGGGATCC
GGGCAGAGCAAAGAGACATGAGGAGCAGGCAGCTCCCAGAGACATGGTGGATTCACGGGAGTGAGGCAGCTTAACTGCCGGAG
GAGACCCAAAGGATGAGCTAGGGAGATCCATCCAAGGGTGGAGAGAGATGAGGGTTCTGGGGAGAAGTGACTCCACTGGAGGGT
GGGAGAGTGTTTAGGAGTGGGAGGGTGGGGGAGGGGAATCCTTGGAAGACCGGGGAGTCATACGGATTGGGAGAAATCCTGGA
AGCAGGGCTGTGGGACCTAAATGTCTGAGTTGATGTACCGCAGTCAAGATATGGCAGAGGCTCCGTGGAAAACTCACTTGGGAGC
AGGGACCCAAAGCAGCAGCCTGAGCTCATGATCAGAGTGAAAGGAGAAGGCTTGTGAGGTCCGTGAATTCCCAGGGCTGAGTTC
ATTCCCTCTGGGGCTGCCCCATACTCATCCCATTACCCCCCCCACCAGCCCTCCCAAAGCCCATGCACACTTCCCAACTCTCAAGC
TGCTCTGCCTTCAGCCACTTCCTCCAAGAACTCAAACAGGGGGCTTTCCCTCCTCAATATCATGTCTCCCCCCTTATGCACCCAGCT
TTCAGAAGCACCCCCCCATGCTAAGTTCTCCCCCATGGATGTCCCATTTAGAAATCAAAAGGAAATAGACACAGGCATGGTCTTTCT
ACAAAGAAACAGACAATGATTAGCTCTGGAGGACAGAGAAGAAATGGGTTTCAGTTCTCAGGGTCCTATACAACACACACACACAC
ACACACACACACACACACACACACACACACCCTCCTGATTGGCCCCAGATTGCCACAGAATCCTGGTGGGGACGACGGGGAGGAGAT
TCCTTGATGCCTGGGTGTCCCCAACTTTCCAAACCCTCTGCCCCCGCGATGGAGAAGAAACCGAGACAGAGGTGTAGGGCCACTA
CCGCTTCCTCCACATGAGATCATGGTTTTCTCCACCAAGGAAGTTTTCCGAGGGTTGAATGAGAGCTTTTCCCCGCCCTCTTCCCC
AAGGGCTATAAAGGCGGCCGTCTGCACAGCCAGCC

mTnfα-mut promoter sequence： 1990bp

TGGAGAAAGCTGCTCCCCCAGGGCCATTCCCACTCCCATCTACCTGGCACAC(del.)G(del.)G(del.)TTTTCCTCCCAATACCCCTTCC
ATGTGCCTCTCCTCAGTGCGCAGAAGTCTGTGTATCCGGGACTTCAAGGACCGTGGGTGCGCTCAATGTACCAGGGGGCTGTGTT
CCTGCTCAGTAAGGGAGACCAGCTGTCCACCCACACCGACGGCATCTCCCATCTACACTTCAGCCCCAGCAGTGTATTCTTTGGAG
CCTTTGCACTGTAGATTCTAAAGAAACCCAAGAATTGGATTCCAGGCCTCCATCCTGACCGTTGTTTCAAGGGTCACATCCCCACAG
TCTCCAGCCTTCCCCACTAAAATAACCTGGAGCTCTCACGGGAGTCTGAGACACTTCAGGGGACTACATCTTCCCCAGGGCCACTC
CAGATGCTCAGGGGACGACTCAAGCCTACCTAGAAGTTCCTGCACAGAGCAGGGTTTTTGTGGGTCTAGGTCGGACAGAGACCTG
GACATGAAGGAGGGACAGACATGGGAGAGGTGGCTGGGAACAGGGGAAGGTTGACTATTTATGGAGAGAAAAGTTAAGTTATTTAT
TTATAGAGAATAGAAAGAGGGGAAAAATAGAAAGCCGTCAGATGACAACTAGGTCCCAGACACAAAGGTGTCTCACCTCAGACAGG
ACCCATCTAAGAGAGAGATGGCGAGAGAATTAGATGTGGGTGACCAAGGGGTTCTAGAAGAAAGCACGAAGCTCTAAAAGCCAGC
CACTGCTTGGCTAGACATCCACAGGGACCCCCTGCACCATCTGTGAAACCCAATAAACCTCTTTTCTCTGAGATTCTGTCTGCTTGT
GTCTGTCTTGCGTTGGGGGAGAAACTTCCTGGTCTCTTTAAGGAGTGGAGCAGGGGACAGAGGCCTCAGTTGGTCCATGGGATCC
GGGCAGAGCAAAGAGACATGAGGAGCAGGCAGCTCCCAGAGACATGGTGGATTCACGGGAGTGAGGCAGCTTAACTGCCGGAG
GAGACCCAAAGGATGAGCTAGGGAGATCCATCCAAGGGTGGAGAGAGATGAGGGTTCTGGGGAGAAGTGACTCCACTGGAGGGT
GGGAGAGTGTTTAGGAGTGGGAGGGTGGGGGAGGGGAATCCTTGGAAGACCGGGGAGTCATACGGATTGGGAGAAATCCTGGA
AGCAGGGCTGTGGGACCTAAATGTCTGAGTTGATGTACCGCAGTCAAGATATGGCAGAGGCTCCGTGGAAAACTCACTTGGGAGC
AGGGACCCAAAGCAGCAGCCTGAGCTCATGATCAGAGTGAAAGGAGAAGGCTTGTGAGGTCCGTGAATTCCCAGGGCTGAGTTC
ATTCCCTCTGGGGCTGCCCCATACTCATCCCATTACCCCCCCCACCAGCCCTCCCAAAGCCCATGCACACTTCCCAACTCTCAAGC
TGCTCTGCCTTCAGCCACTTCCTCCAAGAACTCAAACAGGGGGCTTTCCCTCCTCAATATCATGTCTCCCCCCTTATGCACCCAGCT
TTCAGAAGCACCCCCCCATGCTAAGTTCTCCCCCATGGATGTCCCATTTAGAAATCAAAAGGAAATAGACACAGGCATGGTCTTTCT
ACAAAGAAACAGACAATGATTAGCTCTGGAGGACAGAGAAGAAATGGGTTTCAGTTCTCAGGGTCCTATACAACACACACACACAC
ACACACACACACACACACACACACACACACCCTCCTGATTGGCCCCAGATTGCCACAGAATCCTGGTGGGGACGACGGGGAGGAGAT
TCCTTGATGCCTGGGTGTCCCCAACTTTCCAAACCCTCTGCCCCCGCGATGGAGAAGAAACCGAGACAGAGGTGTAGGGCCACTA
CCGCTTCCTCCACATGAGATCATGGTTTTCTCCACCAAGGAAGTTTTCCGAGGGTTGAATGAGAGCTTTTCCCCGCCCTCTTCCCC
AAGGGCTATAAAGGCGGCCGTCTGCACAGCCAGCC

# Supplemental Table V. The mutant mouse Atf6 sequence

mAtf6-299Arg-mut sequence：1971bp

ATGGAGTCGCCTTTTAGTCCGGTTCTTCCTCATGGACCAGATGAAGACTGGGAGTCTACGTTGTTTGCTGAACTTGGCTATTTCACAGACA
CTGATGATGTGCACTTTGATGCAGCACATGAGGCTTATGAAAATAATTTTGATCATCTTAATTTTGATTTGGATTTGATGCCTTGGGAGTCAG
ACCTATGGAGCCCCGGCAGCCACTTCTGCTCAGACATGAAGGCAGAGCCCCAGCCTCTTTCTCCGGCTTCCTCCAGTTGCTCCATCTCC
TCTCCTCGGTCCACAGACTCGTGTTCTTCAACTCAGCACGTTCCTGAGGAGTTGGATTTGTTGTCTAGTTCTCAGTCCCCCCTTTCCTTAT
ATGGCGACAGCTGTAATAGCCCCTCCTCTGTAGAGCCACTGAAGGAAGAGAAGCCTGTCACTGGTCCTGGAAACAAAACAGAACATGGA
CTGACTCCAAAGAAAAAAATTCAGATGAGTTCAAAACCTTCAGTTCAGCCCAAGCCTTTATTACTTCCAGCAGCGCCCAAGACTCAAACCA
ATGCCAGTGTCCCAGCAAAAGCCATCATCATTCAGACACTACCAGCCCTTATGCCACTGGCAAAGCAGCAGTCGATTATCAGCATACAGCC
TGCGCCCACCAAAGGCCAGACTGTTTTGCTCTCTCAGCCGACTGTGGTTCAACTTCAGAGCCCTGCGGTTCTGTCGTCTGCTCAGCCGG
TTCTCGCAGTCACTGGGGGAGCCGCACAGCTGCCTAACCATGTGGTGAATGTGCTGCCAGCCCCTGTGGTGAGCAGCCCGGTGAATGG
AAAACTCTCCGTGACTAAACCTGTTCTACAAAGTGCCACCAGAAGTATGGGTTCGGATATCGCTGTGCTGAGGAGACAGCAGgctATGATAA
AGAACCGAGAGTCTGCTTGTCAGTCGCGCAAGAAGAAGAAAGAGTATATGCTAGGACTGGAGGCCAGGCTCAAGGCTGCCCTCTCAGAG
AATGAGCAGCTGAAGAAGGAGAATGGCTCCCTGAAGCGACAGCTGGACGAGGTGGTGTCAGAGAACCAGAGGCTCAAAGTCCCAAGTC
CAAAGCGAAGAGCTGTCTGTGTGATGATAGTATTAGCATTTATAATGCTGAACTATGGGCCCATGAGCATGCTGGAGCAAGAGTCCCGAAG
AGTGAAACCTAGTGTGAGCCCTGCCAATCAGAGGAGGCATCTCTTGGAATTTTCAGCGAAAGAAGTTAAAGACACATCCGATGGTGACAA
CCAGAAAGACAGTTACAGCTATGATCACTCTGTGTCCAATGACAAAGCTTTAATGGTGCTAAGTGAAGAGCCATTGCTTTATATGCCTCCAC
CTCCATGTCAACCCCTGATTAACACGACAGAGTCTCTCAGGTTGAACCATGAACTTCGAGGCTGGGTTCATAGACATGAAGTGGAAAGGA
CCAAATCTAGAAGAATGACAAATAGCCAACAGAAAGCCCGCATTCTCCAGGGTGCTCTGGAACAGGGCTCTAATTCTCAGCTGATGGCTG
TCCAGTACACAGAAACCACTAGCATCAGTAGGAATTCTGGGAGTGAGCTGCAAGTGTATTACGCCTCCCCTGGAAGTTACCAAGGCTTCTT
TGACGCCATCCGCAGGAGGGGAGATACGTTTTACGTGGTCTCATTTCGAAGGGATCATCTGCTATTACCAGCTACCACCCACAACAAGAC
CACAAGACCAAAAATGTCAATTGTATTACCAGCAATAAACATAAATGATAATGTGATCAATGGGCAGGACTATGAAGTGATGATGCAGATTGA
CTGTCAGGTGATGGACACCAGGATCCTCCACATCAAAAGCTCCTCAGTCCCCCCTTATCTCCGGGATCATCAGCGGAACCAAACCAGCAC
CTTCTTTGGTTCCCCTCCAACAACCACAGAGACGACCCATGTGGTCAGCACCATCCCTGAGTCGTTGCAGTAG

mAtf6-302Lys-mut sequence：1971bp

ATGGAGTCGCCTTTTAGTCCGGTTCTTCCTCATGGACCAGATGAAGACTGGGAGTCTACGTTGTTTGCTGAACTTGGCTATTTCACAGACA
CTGATGATGTGCACTTTGATGCAGCACATGAGGCTTATGAAAATAATTTTGATCATCTTAATTTTGATTTGGATTTGATGCCTTGGGAGTCAG
ACCTATGGAGCCCCGGCAGCCACTTCTGCTCAGACATGAAGGCAGAGCCCCAGCCTCTTTCTCCGGCTTCCTCCAGTTGCTCCATCTCC
TCTCCTCGGTCCACAGACTCGTGTTCTTCAACTCAGCACGTTCCTGAGGAGTTGGATTTGTTGTCTAGTTCTCAGTCCCCCCTTTCCTTAT
ATGGCGACAGCTGTAATAGCCCCTCCTCTGTAGAGCCACTGAAGGAAGAGAAGCCTGTCACTGGTCCTGGAAACAAAACAGAACATGGA
CTGACTCCAAAGAAAAAAATTCAGATGAGTTCAAAACCTTCAGTTCAGCCCAAGCCTTTATTACTTCCAGCAGCGCCCAAGACTCAAACCA
ATGCCAGTGTCCCAGCAAAAGCCATCATCATTCAGACACTACCAGCCCTTATGCCACTGGCAAAGCAGCAGTCGATTATCAGCATACAGCC
TGCGCCCACCAAAGGCCAGACTGTTTTGCTCTCTCAGCCGACTGTGGTTCAACTTCAGAGCCCTGCGGTTCTGTCGTCTGCTCAGCCGG
TTCTCGCAGTCACTGGGGGAGCCGCACAGCTGCCTAACCATGTGGTGAATGTGCTGCCAGCCCCTGTGGTGAGCAGCCCGGTGAATGG
AAAACTCTCCGTGACTAAACCTGTTCTACAAAGTGCCACCAGAAGTATGGGTTCGGATATCGCTGTGCTGAGGAGACAGCAGCGGATGAT
AgctAACCGAGAGTCTGCTTGTCAGTCGCGCAAGAAGAAGAAAGAGTATATGCTAGGACTGGAGGCCAGGCTCAAGGCTGCCCTCTCAGA
GAATGAGCAGCTGAAGAAGGAGAATGGCTCCCTGAAGCGACAGCTGGACGAGGTGGTGTCAGAGAACCAGAGGCTCAAAGTCCCAAGT
CCAAAGCGAAGAGCTGTCTGTGTGATGATAGTATTAGCATTTATAATGCTGAACTATGGGCCCATGAGCATGCTGGAGCAAGAGTCCCGAA
GAGTGAAACCTAGTGTGAGCCCTGCCAATCAGAGGAGGCATCTCTTGGAATTTTCAGCGAAAGAAGTTAAAGACACATCCGATGGTGACA
ACCAGAAAGACAGTTACAGCTATGATCACTCTGTGTCCAATGACAAAGCTTTAATGGTGCTAAGTGAAGAGCCATTGCTTTATATGCCTCCA
CCTCCATGTCAACCCCTGATTAACACGACAGAGTCTCTCAGGTTGAACCATGAACTTCGAGGCTGGGTTCATAGACATGAAGTGGAAAGG
ACCAAATCTAGAAGAATGACAAATAGCCAACAGAAAGCCCGCATTCTCCAGGGTGCTCTGGAACAGGGCTCTAATTCTCAGCTGATGGCT
GTCCAGTACACAGAAACCACTAGCATCAGTAGGAATTCTGGGAGTGAGCTGCAAGTGTATTACGCCTCCCCTGGAAGTTACCAAGGCTTC
TTTGACGCCATCCGCAGGAGGGGAGATACGTTTTACGTGGTCTCATTTCGAAGGGATCATCTGCTATTACCAGCTACCACCCACAACAAGA
CCACAAGACCAAAAATGTCAATTGTATTACCAGCAATAAACATAAATGATAATGTGATCAATGGGCAGGACTATGAAGTGATGATGCAGATTG
ACTGTCAGGTGATGGACACCAGGATCCTCCACATCAAAAGCTCCTCAGTCCCCCCTTATCTCCGGGATCATCAGCGGAACCAAACCAGCA
CCTTCTTTGGTTCCCCTCCAACAACCACAGAGACGACCCATGTGGTCAGCACCATCCCTGAGTCGTTGCAGTAG

mAtf6-303Asn-mut sequence：1971bp

ATGGAGTCGCCTTTTAGTCCGGTTCTTCCTCATGGACCAGATGAAGACTGGGAGTCTACGTTGTTTGCTGAACTTGGCTATTTCACAGACA
CTGATGATGTGCACTTTGATGCAGCACATGAGGCTTATGAAAATAATTTTGATCATCTTAATTTTGATTTGGATTTGATGCCTTGGGAGTCAG
ACCTATGGAGCCCCGGCAGCCACTTCTGCTCAGACATGAAGGCAGAGCCCCAGCCTCTTTCTCCGGCTTCCTCCAGTTGCTCCATCTCC
TCTCCTCGGTCCACAGACTCGTGTTCTTCAACTCAGCACGTTCCTGAGGAGTTGGATTTGTTGTCTAGTTCTCAGTCCCCCCTTTCCTTAT
ATGGCGACAGCTGTAATAGCCCCTCCTCTGTAGAGCCACTGAAGGAAGAGAAGCCTGTCACTGGTCCTGGAAACAAAACAGAACATGGA
CTGACTCCAAAGAAAAAAATTCAGATGAGTTCAAAACCTTCAGTTCAGCCCAAGCCTTTATTACTTCCAGCAGCGCCCAAGACTCAAACCA
ATGCCAGTGTCCCAGCAAAAGCCATCATCATTCAGACACTACCAGCCCTTATGCCACTGGCAAAGCAGCAGTCGATTATCAGCATACAGCC
TGCGCCCACCAAAGGCCAGACTGTTTTGCTCTCTCAGCCGACTGTGGTTCAACTTCAGAGCCCTGCGGTTCTGTCGTCTGCTCAGCCGG
TTCTCGCAGTCACTGGGGGAGCCGCACAGCTGCCTAACCATGTGGTGAATGTGCTGCCAGCCCCTGTGGTGAGCAGCCCGGTGAATGG
AAAACTCTCCGTGACTAAACCTGTTCTACAAAGTGCCACCAGAAGTATGGGTTCGGATATCGCTGTGCTGAGGAGACAGCAGCGGATGAT
AAAGgctCGAGAGTCTGCTTGTCAGTCGCGCAAGAAGAAGAAAGAGTATATGCTAGGACTGGAGGCCAGGCTCAAGGCTGCCCTCTCAGA
GAATGAGCAGCTGAAGAAGGAGAATGGCTCCCTGAAGCGACAGCTGGACGAGGTGGTGTCAGAGAACCAGAGGCTCAAAGTCCCAAGT
CCAAAGCGAAGAGCTGTCTGTGTGATGATAGTATTAGCATTTATAATGCTGAACTATGGGCCCATGAGCATGCTGGAGCAAGAGTCCCGAA
GAGTGAAACCTAGTGTGAGCCCTGCCAATCAGAGGAGGCATCTCTTGGAATTTTCAGCGAAAGAAGTTAAAGACACATCCGATGGTGACA
ACCAGAAAGACAGTTACAGCTATGATCACTCTGTGTCCAATGACAAAGCTTTAATGGTGCTAAGTGAAGAGCCATTGCTTTATATGCCTCCA
CCTCCATGTCAACCCCTGATTAACACGACAGAGTCTCTCAGGTTGAACCATGAACTTCGAGGCTGGGTTCATAGACATGAAGTGGAAAGG
ACCAAATCTAGAAGAATGACAAATAGCCAACAGAAAGCCCGCATTCTCCAGGGTGCTCTGGAACAGGGCTCTAATTCTCAGCTGATGGCT
GTCCAGTACACAGAAACCACTAGCATCAGTAGGAATTCTGGGAGTGAGCTGCAAGTGTATTACGCCTCCCCTGGAAGTTACCAAGGCTTC
TTTGACGCCATCCGCAGGAGGGGAGATACGTTTTACGTGGTCTCATTTCGAAGGGATCATCTGCTATTACCAGCTACCACCCACAACAAGA
CCACAAGACCAAAAATGTCAATTGTATTACCAGCAATAAACATAAATGATAATGTGATCAATGGGCAGGACTATGAAGTGATGATGCAGATTG
ACTGTCAGGTGATGGACACCAGGATCCTCCACATCAAAAGCTCCTCAGTCCCCCCTTATCTCCGGGATCATCAGCGGAACCAAACCAGCA
CCTTCTTTGGTTCCCCTCCAACAACCACAGAGACGACCCATGTGGTCAGCACCATCCCTGAGTCGTTGCAGTAG

mAtf6-304Arg-mut sequence：1971bp

ATGGAGTCGCCTTTTAGTCCGGTTCTTCCTCATGGACCAGATGAAGACTGGGAGTCTACGTTGTTTGCTGAACTTGGCTATTTCACAGACA
CTGATGATGTGCACTTTGATGCAGCACATGAGGCTTATGAAAATAATTTTGATCATCTTAATTTTGATTTGGATTTGATGCCTTGGGAGTCAG
ACCTATGGAGCCCCGGCAGCCACTTCTGCTCAGACATGAAGGCAGAGCCCCAGCCTCTTTCTCCGGCTTCCTCCAGTTGCTCCATCTCC
TCTCCTCGGTCCACAGACTCGTGTTCTTCAACTCAGCACGTTCCTGAGGAGTTGGATTTGTTGTCTAGTTCTCAGTCCCCCCTTTCCTTAT
ATGGCGACAGCTGTAATAGCCCCTCCTCTGTAGAGCCACTGAAGGAAGAGAAGCCTGTCACTGGTCCTGGAAACAAAACAGAACATGGA
CTGACTCCAAAGAAAAAAATTCAGATGAGTTCAAAACCTTCAGTTCAGCCCAAGCCTTTATTACTTCCAGCAGCGCCCAAGACTCAAACCA
ATGCCAGTGTCCCAGCAAAAGCCATCATCATTCAGACACTACCAGCCCTTATGCCACTGGCAAAGCAGCAGTCGATTATCAGCATACAGCC
TGCGCCCACCAAAGGCCAGACTGTTTTGCTCTCTCAGCCGACTGTGGTTCAACTTCAGAGCCCTGCGGTTCTGTCGTCTGCTCAGCCGG
TTCTCGCAGTCACTGGGGGAGCCGCACAGCTGCCTAACCATGTGGTGAATGTGCTGCCAGCCCCTGTGGTGAGCAGCCCGGTGAATGG
AAAACTCTCCGTGACTAAACCTGTTCTACAAAGTGCCACCAGAAGTATGGGTTCGGATATCGCTGTGCTGAGGAGACAGCAGCGGATGAT
AAAGAACgctGAGTCTGCTTGTCAGTCGCGCAAGAAGAAGAAAGAGTATATGCTAGGACTGGAGGCCAGGCTCAAGGCTGCCCTCTCAGA
GAATGAGCAGCTGAAGAAGGAGAATGGCTCCCTGAAGCGACAGCTGGACGAGGTGGTGTCAGAGAACCAGAGGCTCAAAGTCCCAAGT
CCAAAGCGAAGAGCTGTCTGTGTGATGATAGTATTAGCATTTATAATGCTGAACTATGGGCCCATGAGCATGCTGGAGCAAGAGTCCCGAA
GAGTGAAACCTAGTGTGAGCCCTGCCAATCAGAGGAGGCATCTCTTGGAATTTTCAGCGAAAGAAGTTAAAGACACATCCGATGGTGACA
ACCAGAAAGACAGTTACAGCTATGATCACTCTGTGTCCAATGACAAAGCTTTAATGGTGCTAAGTGAAGAGCCATTGCTTTATATGCCTCCA
CCTCCATGTCAACCCCTGATTAACACGACAGAGTCTCTCAGGTTGAACCATGAACTTCGAGGCTGGGTTCATAGACATGAAGTGGAAAGG
ACCAAATCTAGAAGAATGACAAATAGCCAACAGAAAGCCCGCATTCTCCAGGGTGCTCTGGAACAGGGCTCTAATTCTCAGCTGATGGCT
GTCCAGTACACAGAAACCACTAGCATCAGTAGGAATTCTGGGAGTGAGCTGCAAGTGTATTACGCCTCCCCTGGAAGTTACCAAGGCTTC
TTTGACGCCATCCGCAGGAGGGGAGATACGTTTTACGTGGTCTCATTTCGAAGGGATCATCTGCTATTACCAGCTACCACCCACAACAAGA
CCACAAGACCAAAAATGTCAATTGTATTACCAGCAATAAACATAAATGATAATGTGATCAATGGGCAGGACTATGAAGTGATGATGCAGATTG
ACTGTCAGGTGATGGACACCAGGATCCTCCACATCAAAAGCTCCTCAGTCCCCCCTTATCTCCGGGATCATCAGCGGAACCAAACCAGCA
CCTTCTTTGGTTCCCCTCCAACAACCACAGAGACGACCCATGTGGTCAGCACCATCCCTGAGTCGTTGCAGTAG

mAtf6-311Arg-mut sequence：1971bp

ATGGAGTCGCCTTTTAGTCCGGTTCTTCCTCATGGACCAGATGAAGACTGGGAGTCTACGTTGTTTGCTGAACTTGGCTATTTCACAGACA
CTGATGATGTGCACTTTGATGCAGCACATGAGGCTTATGAAAATAATTTTGATCATCTTAATTTTGATTTGGATTTGATGCCTTGGGAGTCAG
ACCTATGGAGCCCCGGCAGCCACTTCTGCTCAGACATGAAGGCAGAGCCCCAGCCTCTTTCTCCGGCTTCCTCCAGTTGCTCCATCTCC
TCTCCTCGGTCCACAGACTCGTGTTCTTCAACTCAGCACGTTCCTGAGGAGTTGGATTTGTTGTCTAGTTCTCAGTCCCCCCTTTCCTTAT
ATGGCGACAGCTGTAATAGCCCCTCCTCTGTAGAGCCACTGAAGGAAGAGAAGCCTGTCACTGGTCCTGGAAACAAAACAGAACATGGA
CTGACTCCAAAGAAAAAAATTCAGATGAGTTCAAAACCTTCAGTTCAGCCCAAGCCTTTATTACTTCCAGCAGCGCCCAAGACTCAAACCA
ATGCCAGTGTCCCAGCAAAAGCCATCATCATTCAGACACTACCAGCCCTTATGCCACTGGCAAAGCAGCAGTCGATTATCAGCATACAGCC
TGCGCCCACCAAAGGCCAGACTGTTTTGCTCTCTCAGCCGACTGTGGTTCAACTTCAGAGCCCTGCGGTTCTGTCGTCTGCTCAGCCGG
TTCTCGCAGTCACTGGGGGAGCCGCACAGCTGCCTAACCATGTGGTGAATGTGCTGCCAGCCCCTGTGGTGAGCAGCCCGGTGAATGG
AAAACTCTCCGTGACTAAACCTGTTCTACAAAGTGCCACCAGAAGTATGGGTTCGGATATCGCTGTGCTGAGGAGACAGCAGCGGATGAT
AAAGAACCGAGAGTCTGCTTGTCAGTCGgctAAGAAGAAGAAAGAGTATATGCTAGGACTGGAGGCCAGGCTCAAGGCTGCCCTCTCAGA
GAATGAGCAGCTGAAGAAGGAGAATGGCTCCCTGAAGCGACAGCTGGACGAGGTGGTGTCAGAGAACCAGAGGCTCAAAGTCCCAAGT
CCAAAGCGAAGAGCTGTCTGTGTGATGATAGTATTAGCATTTATAATGCTGAACTATGGGCCCATGAGCATGCTGGAGCAAGAGTCCCGAA
GAGTGAAACCTAGTGTGAGCCCTGCCAATCAGAGGAGGCATCTCTTGGAATTTTCAGCGAAAGAAGTTAAAGACACATCCGATGGTGACA
ACCAGAAAGACAGTTACAGCTATGATCACTCTGTGTCCAATGACAAAGCTTTAATGGTGCTAAGTGAAGAGCCATTGCTTTATATGCCTCCA
CCTCCATGTCAACCCCTGATTAACACGACAGAGTCTCTCAGGTTGAACCATGAACTTCGAGGCTGGGTTCATAGACATGAAGTGGAAAGG
ACCAAATCTAGAAGAATGACAAATAGCCAACAGAAAGCCCGCATTCTCCAGGGTGCTCTGGAACAGGGCTCTAATTCTCAGCTGATGGCT
GTCCAGTACACAGAAACCACTAGCATCAGTAGGAATTCTGGGAGTGAGCTGCAAGTGTATTACGCCTCCCCTGGAAGTTACCAAGGCTTC
TTTGACGCCATCCGCAGGAGGGGAGATACGTTTTACGTGGTCTCATTTCGAAGGGATCATCTGCTATTACCAGCTACCACCCACAACAAGA
CCACAAGACCAAAAATGTCAATTGTATTACCAGCAATAAACATAAATGATAATGTGATCAATGGGCAGGACTATGAAGTGATGATGCAGATTG
ACTGTCAGGTGATGGACACCAGGATCCTCCACATCAAAAGCTCCTCAGTCCCCCCTTATCTCCGGGATCATCAGCGGAACCAAACCAGCA
CCTTCTTTGGTTCCCCTCCAACAACCACAGAGACGACCCATGTGGTCAGCACCATCCCTGAGTCGTTGCAGTAG

mAtf6-315Lys-mut sequence：1971bp

ATGGAGTCGCCTTTTAGTCCGGTTCTTCCTCATGGACCAGATGAAGACTGGGAGTCTACGTTGTTTGCTGAACTTGGCTATTTCACAGACA
CTGATGATGTGCACTTTGATGCAGCACATGAGGCTTATGAAAATAATTTTGATCATCTTAATTTTGATTTGGATTTGATGCCTTGGGAGTCAG
ACCTATGGAGCCCCGGCAGCCACTTCTGCTCAGACATGAAGGCAGAGCCCCAGCCTCTTTCTCCGGCTTCCTCCAGTTGCTCCATCTCC
TCTCCTCGGTCCACAGACTCGTGTTCTTCAACTCAGCACGTTCCTGAGGAGTTGGATTTGTTGTCTAGTTCTCAGTCCCCCCTTTCCTTAT
ATGGCGACAGCTGTAATAGCCCCTCCTCTGTAGAGCCACTGAAGGAAGAGAAGCCTGTCACTGGTCCTGGAAACAAAACAGAACATGGA
CTGACTCCAAAGAAAAAAATTCAGATGAGTTCAAAACCTTCAGTTCAGCCCAAGCCTTTATTACTTCCAGCAGCGCCCAAGACTCAAACCA
ATGCCAGTGTCCCAGCAAAAGCCATCATCATTCAGACACTACCAGCCCTTATGCCACTGGCAAAGCAGCAGTCGATTATCAGCATACAGCC
TGCGCCCACCAAAGGCCAGACTGTTTTGCTCTCTCAGCCGACTGTGGTTCAACTTCAGAGCCCTGCGGTTCTGTCGTCTGCTCAGCCGG
TTCTCGCAGTCACTGGGGGAGCCGCACAGCTGCCTAACCATGTGGTGAATGTGCTGCCAGCCCCTGTGGTGAGCAGCCCGGTGAATGG
AAAACTCTCCGTGACTAAACCTGTTCTACAAAGTGCCACCAGAAGTATGGGTTCGGATATCGCTGTGCTGAGGAGACAGCAGCGGATGAT
AAAGAACCGAGAGTCTGCTTGTCAGTCGCGCAAGAAGAAGgctGAGTATATGCTAGGACTGGAGGCCAGGCTCAAGGCTGCCCTCTCAGA
GAATGAGCAGCTGAAGAAGGAGAATGGCTCCCTGAAGCGACAGCTGGACGAGGTGGTGTCAGAGAACCAGAGGCTCAAAGTCCCAAGT
CCAAAGCGAAGAGCTGTCTGTGTGATGATAGTATTAGCATTTATAATGCTGAACTATGGGCCCATGAGCATGCTGGAGCAAGAGTCCCGAA
GAGTGAAACCTAGTGTGAGCCCTGCCAATCAGAGGAGGCATCTCTTGGAATTTTCAGCGAAAGAAGTTAAAGACACATCCGATGGTGACA
ACCAGAAAGACAGTTACAGCTATGATCACTCTGTGTCCAATGACAAAGCTTTAATGGTGCTAAGTGAAGAGCCATTGCTTTATATGCCTCCA
CCTCCATGTCAACCCCTGATTAACACGACAGAGTCTCTCAGGTTGAACCATGAACTTCGAGGCTGGGTTCATAGACATGAAGTGGAAAGG
ACCAAATCTAGAAGAATGACAAATAGCCAACAGAAAGCCCGCATTCTCCAGGGTGCTCTGGAACAGGGCTCTAATTCTCAGCTGATGGCT
GTCCAGTACACAGAAACCACTAGCATCAGTAGGAATTCTGGGAGTGAGCTGCAAGTGTATTACGCCTCCCCTGGAAGTTACCAAGGCTTC
TTTGACGCCATCCGCAGGAGGGGAGATACGTTTTACGTGGTCTCATTTCGAAGGGATCATCTGCTATTACCAGCTACCACCCACAACAAGA
CCACAAGACCAAAAATGTCAATTGTATTACCAGCAATAAACATAAATGATAATGTGATCAATGGGCAGGACTATGAAGTGATGATGCAGATTG
ACTGTCAGGTGATGGACACCAGGATCCTCCACATCAAAAGCTCCTCAGTCCCCCCTTATCTCCGGGATCATCAGCGGAACCAAACCAGCA
CCTTCTTTGGTTCCCCTCCAACAACCACAGAGACGACCCATGTGGTCAGCACCATCCCTGAGTCGTTGCAGTAG
